# The *Rhizoctonia solani* AG1-IB (isolate 7/3/14) transcriptome during interaction with the host plant lettuce (*Lactuca sativa* L.)

**DOI:** 10.1371/journal.pone.0177278

**Published:** 2017-05-09

**Authors:** Bart Verwaaijen, Daniel Wibberg, Magdalena Kröber, Anika Winkler, Rita Zrenner, Hanna Bednarz, Karsten Niehaus, Rita Grosch, Alfred Pühler, Andreas Schlüter

**Affiliations:** 1 Center for Biotechnology, Bielefeld University, Bielefeld, Germany; 2 Leibniz-Institute of Vegetable and Ornamental Crops (IGZ), Großbeeren, Germany; Wageningen Universiteit en Researchcentrum, NETHERLANDS

## Abstract

The necrotrophic pathogen *Rhizoctonia solani* is one of the most economically important soil-borne pathogens of crop plants. Isolates of *R*. *solani* AG1-IB are the major pathogens responsible for bottom-rot of lettuce (*Lactuca sativa* L.) and are also responsible for diseases in other plant species. Currently, there is lack of information regarding the molecular responses in *R*. *solani* during the pathogenic interaction between the necrotrophic soil-borne pathogen and its host plant. The genome of *R*. *solani* AG1-IB (isolate 7/3/14) was recently established to obtain insights into its putative pathogenicity determinants. In this study, the transcriptional activity of *R*. *solani* AG1-IB was followed during the course of its pathogenic interaction with the host plant lettuce under controlled conditions. Based on visual observations, three distinct pathogen-host interaction zones on lettuce leaves were defined which covered different phases of disease progression on tissue inoculated with the AG1-IB (isolate 7/3/14). The zones were defined as: Zone 1—symptomless, Zone 2—light brown discoloration, and Zone 3—dark brown, necrotic lesions. Differences in *R*. *solani* hyphae structure in these three zones were investigated by microscopic observation. Transcriptional activity within these three interaction zones was used to represent the course of *R*. *solani* disease progression applying high-throughput RNA sequencing (RNA-Seq) analysis of samples collected from each Zone. The resulting three transcriptome data sets were analyzed for their highest expressed genes and for differentially transcribed genes between the respective interaction zones. Among the highest expressed genes was a group of not previously described genes which were transcribed exclusively during early stages of interaction, in Zones 1 and 2. Previously described importance of up-regulation in *R*. *solani* agglutinin genes during disease progression could be further confirmed; here, the corresponding genes exhibited extremely high transcription levels. Most differentially higher expressed transcripts were found within Zone 2. In Zone 3, the zone with the strongest degree of interaction, gene transcripts indicative of apoptotic activity were highly abundant. The transcriptome data presented in this work support previous models of the disease and interaction cycle of *R*. *solani* and lettuce and may influence effective techniques for control of this pathogen.

## Introduction

The soil-borne fungus of the phylum *Basidiomycota*, *Rhizoctonia solani* (teleomorph *Thanatephorus cucumeris* [Frank] Donk) is a necrotrophic plant pathogen. It is recognized as a worldwide pathogen to numerous plant species including the economically important crops rice, soybean, potato, maize, sugar beet, cabbage, cauliflower, tomato, and lettuce [1,2]. There are 14 distinct anastomosis groups (AGs) of *R*. *solani* species, some of which are subdivided into additional subgroups that can be distinguished based on genetic characteristics and ecological criteria, such as specific host range [3]. AG1 is divided into different subgroups, each featuring distinct host specificities. Members of the subgroup AG1-IB are able to infect lettuce (*Lactuca sativa* L.) among other host plants and are the predominant cause of bottom rot on lettuce. *R*. *solani* hyphae enter a plant host via specialized infection hyphae or infection structures, namely infection cushions and/or lobate appressoria. Penetration of the cuticle and epidermal cell wall may be achieved by mechanical pressure, osmotic pressure, enzymatic digestion, or a combination of these mechanisms [4]. The bottom rot pathogen *R*. *solani* AG1-IB initially infects lower leaves of lettuce that are in contact to the soil surface. Small rust-colored to dark brown spots appear on lower midribs; later, leaf lesions and withered outer wrapper leaves are formed [5]. The pathogen can spread rapidly by hyphae on leaf surfaces into the lettuce head causing rot of midribs and leaves [6]. In later stages of bottom rot, dark brown sclerotia occur on leaves. The pathogen survives in the soil as sclerotia or as melanized mycelium which can be associated with organic debris until a new disease cycle is initiated.

During pathogenesis, necrotrophs secrete compounds including phytotoxins, cell wall degrading enzymes, and other extracellular enzymes affecting host tissue. These may represent pathogenicity determinants for the host species [7,8]. Secretion of these compounds occurs prior to and during colonization including primary infection and results in formation and enlargement of lesions [9]. After appearance of initial necrosis, further disease progression culminates in death and decay of the entire plant. A lack of knowledge exists regarding secreted compounds during pathogenesis of *R*. *solani* AG1-IB, since they have not been specified for particular host plants such as lettuce.

A major step forward towards increased understanding of this plant pathogen was achieved by sequencing of the *R*. *solani* AG1-IB (isolate 7/3/14) genome and the subsequent sequencing of an Expressed Sequence Tags (ESTs) library [10–12]. In response to lettuce root exudates, many ESTs of *R*. *solani* AG1-IB were predicted to encode plant cell wall degrading enzymes such as cellulases, pectinases and ligninases, as well as phytotoxins [11]. Furthermore, some *R*. *solani* AG1-IB ESTs were coupled to the suppression of the plant defense response [11].

Dual RNA-Seq is a new approach that enables access to the transcriptomes of two or more interacting organisms simultaneously [13]. Taking advantage of this method, we present first results of a high coverage dual RNA-Seq experiment of *R*. *solani* AG1-IB (isolate 7/3/14) in interaction with lettuce leaves with focus on the *R*. *solani* transcriptome. During the early phase of interspecific interaction, we especially expect the expression of genes related to suppression of the plant’s defense response accompanied by secretion of phytotoxins resulting in induction of necrosis. In the further advanced phases of interaction, plant compounds serve as nutrient source for the fungus. Therefore, we assume that the majority of expressed genes are related to the metabolization of these compounds. We postulated that during its infection cycle, the pathogen *R*. *solani* AG1-IB would express more genes than the previously known genes of plant cell wall degrading enzymes and that expression profiles of cellular transcripts would vary depending on the stage of the infection cycle. The objective of this investigation is to gain insight into the gene expression in *R*. *solani* during an infection cycle. Using the model patho-system of lettuce and *R*. *solani* AG1-IB, we aimed at determining genes that are of pivotal importance for pathogenic interaction and may be defined as putative pathogenicity determinants of *R*. *solani* AG1-IB.

## Methods

### Cultivation of lettuce

The lettuce cultivar Tizian (Syngenta Seeds GmbH) was cultivated under greenhouse conditions at the University of Bielefeld (Germany). As substrate, a 1:1 volume ratio of quartz sand and Archut Fruhstorfer Einheitserde type N (Hawita gruppe GmbH, Germany) was used and plants were grown in one liter pots. The plants were fertilized weekly with a 0.1% Wuxal Top N solution (Wilhelm Haug GmbH & Co. KG, Germany). During the remainder of the week, only water was provided as required. The greenhouse was maintained at an average temperature of 18°C during the cultivation period, the plants were exposed to natural light supplemented with artificial light (400 watt SON-T Agro Philips) on a 16/8 h day/night regime.

### *R*. *solani* and lettuce interaction

**Fig 1:**
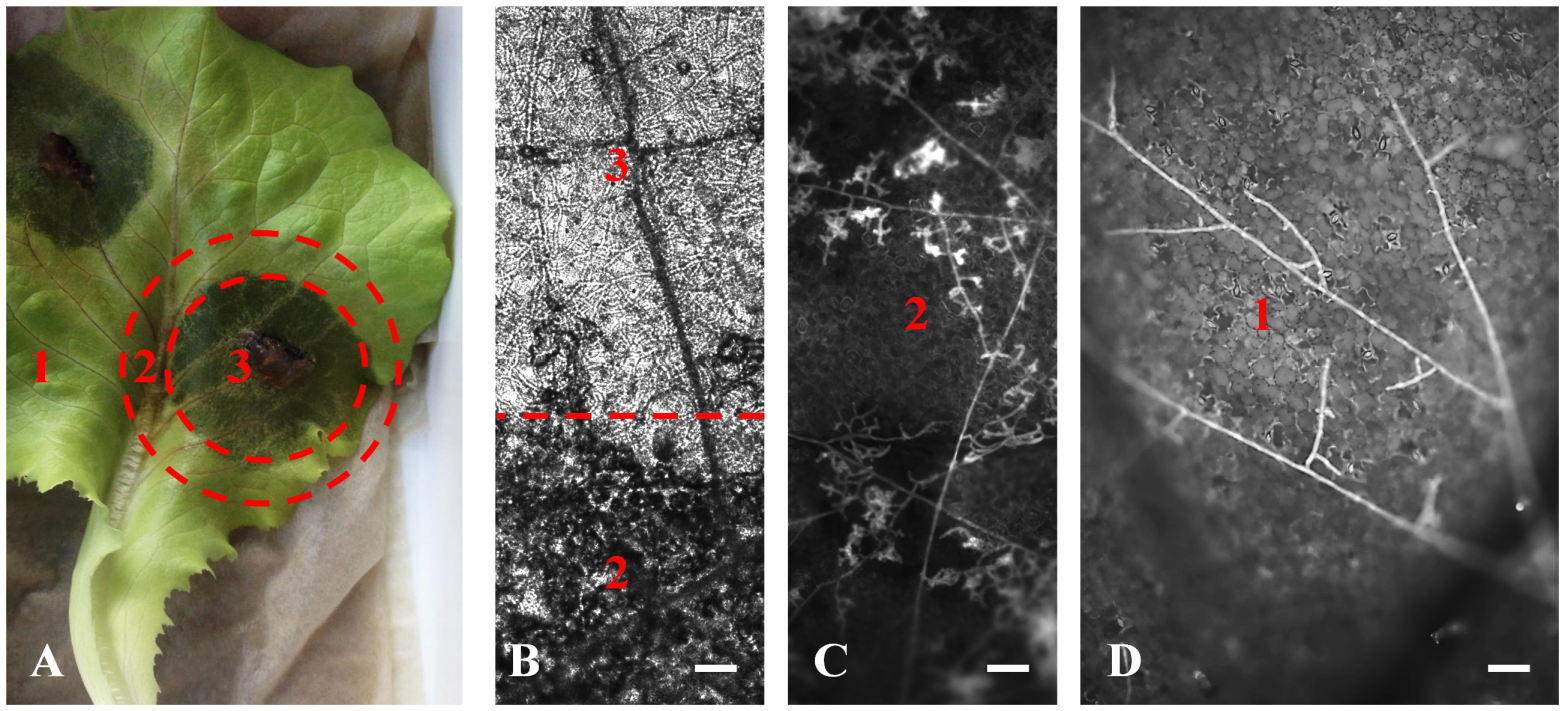
Epifluorescence microscopy of the three interaction zones 1 to 3. (A) Overview microscopic picture depicting all three interaction zones. In Zone 1 specialized infection structures have not been formed and the lettuce cells appear symptomless, in contrast to Zone 2. In the third interaction zone, all lettuce cells appear to have been destroyed. (B) Bright-field microscopic image of Zones 3 and 2. (C) UV-microscopic image of Zone 2 using a GFP filter. (D) UV-microscopic image of Zone 1 using a DAPI filter. The scale bar represents 50 μm.

The fungus *R*. *solani* AG1-IB (isolate 7/3/14) (IGZ, Groβbeeren) was cultured on Difco Potato dextrose agar (Becton Dickinson, Heidelberg, Germany) at room temperature. For the inoculation of lettuce leaves, five day old mycelia disks of approximately 5–7 mm were used. From three lettuce plants with mature heads (cultivated for 12 weeks), nine leaves from each plant were placed in plastic containers with a layer of sterile agarose on the bottom. From each set of leaves, eight were inoculated with a *R*. *solani* mycelia disk. The remaining leaves functioned as blanks (non-inoculated control). During incubation, the containers were loosely covered with clear plastic in order to retain moisture and allow for light transmission. Leaves were incubated for three days at room temperature. Subsequently, samples were taken from three different zones which were used to represent distinct stages of interaction between plant and pathogen. Samples from the eight inoculated leaves were pooled per plant of origin and per interaction zone. Interaction Zone 3 is characterised by dark brown and necrotic lesions around the inoculation site, in Zone 2 light brown lesions are to be observed, whereas the leaf tissue appears still symptomless in Zone 1 (Fig 1A). After flash freezing in liquid nitrogen, samples were stored at -80°C prior to further processing. The samples were ground to a fine powder. Per sample, the equivalent of 0.75 ml of powder was used for total RNA isolation with the RNeasy plant mini kit (Qiagen GmbH, Hilden, Germany). 2.25 μg of RNA per sample was used for library preparation with the TruSeq^®^ mRNA Sample Preparation Kit (stranded) (Illumina Inc., San Diego, U.S.A.).

### Sequencing of cDNA libraries

Sequencing of the prepared cDNA libraries was carried out on the Illumina HiSeq 1500 platform (Illumina Inc., San Diego, U.S.A.). To enable sufficient transcriptome coverage, the cDNA library was sequenced in three runs. The first two runs of the 12 cDNA libraries were sequenced in single read and rapid mode with 67 cycles, whereas in the third run, the samples were paired-end sequenced in high output mode with 2 × 28 cycles. Data analysis and base calling were accomplished with in-house software based on CASAVA 1.8.2. (Illumina). The sequencing raw data for all libraries has been made available on the EBI ArrayExpress server, accession E-MTAB-4762.

### Mapping of short transcriptome reads to the *R*. *solani* AG1-IB reference genome

The sequenced reads were quality filtered (> Q30) by applying the FASTX tool kit [14]. Data of all three replicates of each condition were joined together and subsequently mapped to the improved *R*. *solani* AG1-IB draft genome [15] [EMBL: LN679100 –LN679996] using tophat2 [16]. Two mismatches were allowed to account for possible sequencing errors and allelic variants of the diploid *R*. *solani* AG1-IB genome [10]. The RNA-Seq analysis platform ReadXplorer [17] was used for the visualization of short read alignments.

### Gene expression analysis

Results of the short read mapping were imported into the platform ReadXplorer 2.0.1. [17,18]. The R package DESeq [19] was selected in this work. Genes with a minimum fold change of log_2_ 1, and a p-adjusted value smaller than 0.05 were deemed significant.

Additionally, reads per kilobase per million reads (RPKM) values were calculated from exported read count tables, using the single best match option for each of the separate libraries [20]. Per biological replicate, the values were summed and the RPKM value was calculated based on the standard equation [20]. Probability calculations were omitted for the RPKM calculation, as these values were not used to determine differential significant differences between the datasets, but merely depict general levels of transcription.

### Detailed annotation of highly- and differentially-expressed genes

Selected genes based on differential expression and RPKM results were annotated in detail by several approaches. First, PFAM, KOG and GO annotation was performed by means of WebMGA [21] and the BLAST2GO freeware version 3.1.3. [22] using default settings. In addition, these genes were screened for CAZymes by applying dbCAN [23]. For the identification of cytochrome p450 monooxygenase and multicopper oxidases, BioCatNet (biocatnet.de) CYPED and LccED databases provided by the University of Stuttgart (Stuttgart, Germany) were used. Finally, the Pathogen Host Interaction database (PHI-base) was used to find sequence homology in comparison with known virulence and pathogenicity markers based on BLASTp with a maximum E-value cut-off of 1*10^−20^ [24].

Amino acid sequences of selected genes were extracted from corresponding database entries and analyzed by using MUSCLE and the Neighbor-Joining algorithm implemented in MEGA 6.0 [25–28]. A bootstrap analysis using 1000 re-samplings of the sequence data was carried out.

## Results and discussion

### The interspecific interaction between *R*. *solani* and lettuce

To study the interspecific interaction between *R*. *solani* and lettuce, a leaf model was applied for dual transcriptional analyses. In comparison to a whole plant assay, the leaf model ensured the lowest possible technical and biological variance during fungal-host interaction, as incubation conditions could be more strictly controlled and replicated. During the course of plant-pathogen interaction, visually distinguishable zones appeared around the inoculation site. Dark brown, necrotic lesions occurred next to the inoculation site (Zone 3). Loss of tissue integrity at this stage is presumably caused by the degradation of complex polymers like cellulose, lignin and other structurally important compounds. This Zone was followed by a ring of light brown color (Zone 2). Distal to the latter ring, a zone without direct visual symptoms was selected (Zone 1) (Fig 1A).

**Fig 2:**
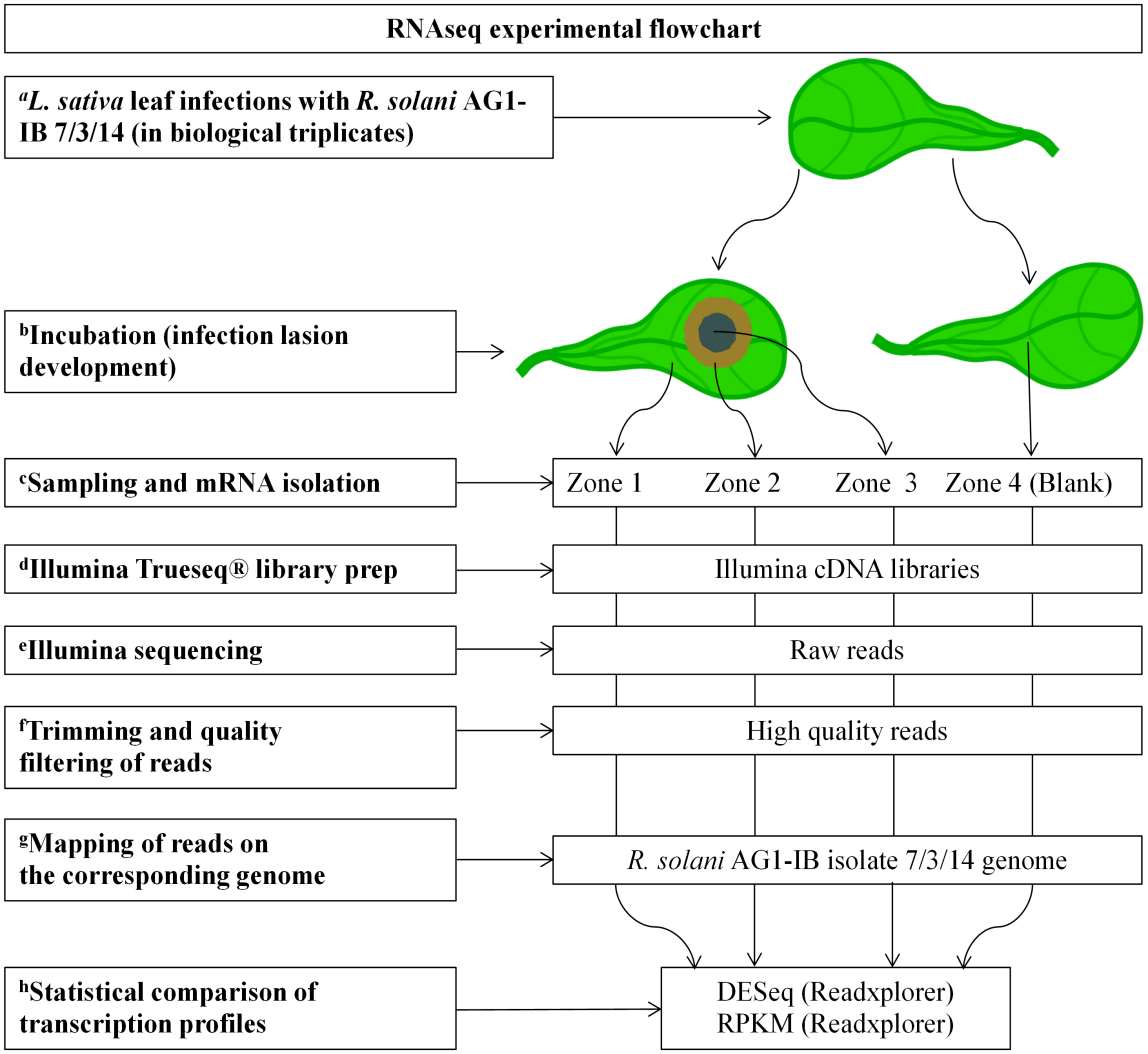
Complete workflow of the experiment. (a) Leaves were taken from three different mature lettuce plants (cv. Tizian) and infected with *R*. *solani* AG1-IB (isolate 7/3/14) mycelia disks; non-infected leaves served as control. (b) During incubation, the *R*. *solani* infected leaves developed lesions. (c) For each of the three biological replicates, three separate interaction zones and the control zone (named Zone 4) were sampled and mRNA was isolated. (d) cDNA libraries were generated applying the Illumina TruSeq mRNA Sample Preparation Kit (stranded). (e) Illumina HiSeq 1500 sequencing. (f) Trimming and quality filtering of raw reads. (g) Mapping of trimmed and quality filtered reads onto the *R*. *solani* AG1-IB (isolate 7/3/14) reference genome sequence. (h) Statistical comparison of transcription profiles by means of DESeq and RPKM value calculations implemented in the ReadXplorer platform [17,18].

Microscopic investigations were made to analyze whether the pathogen forms distinct infection structures such as infection cushions or appressoria prior to infection. This method was also used to determine whether differences in *R*. *solani* hyphae structures in Zones 1 to 3 can be observed (Fig 1B–1D). *R*. *solani* exhibits auto-fluorescence. Therefore, it was possible to perform UV and laser scanning microscopy of hyphal structures. Branching of *R*. *solani* runner hyphae spreading over the leaf surface was visible at the microscopic level prior to infection of lettuce tissue (Zone 1, Fig 1D). No disease symptoms occurred in this zone. Numerous infection structures were formed by *R*. *solani* in Zone 2 (Fig 1C). The formation of these structures is similar to those of early stage infection cushions and lobate appressoria described for *R*. *solani* on rice [29] or *Sclerotinia* sp. on pea [30]. The appearance of necrosis of lettuce cells surrounding these infection structures is regarded as evidence for successful infection and induction of necrosis by the pathogen. This has been postulated to occur through suppression of the natural defense response of the plant *via* secretion of as yet unidentified compounds by the fungus. Here, it was observed that chlorophyll fluorescence was degraded around the infection structures in Zone 2 which was verified by means of laser scanning microscopy (data not shown). At Zone 3, no intact lettuce cells remained and dense *R*. *solani* mycelium sheets were observed (Fig 1B). The lettuce cells seem to be already macerated by the pathogen. The reported interaction zones are similar to those described for fungal phytopathogenic infection models e.g. *Fusarium graminearum*, the causal agent of Fusarium head blight on wheat [31]. Fig 2 summarizes the complete workflow of the experiment, from sampling to analysis of the RNA-sequencing results.

### *R*. *solani* transcriptional profiling covering three interspecific interaction zones as revealed by RNA sequencing

Understanding host-pathogen interactions requires knowledge of transcriptional changes for both interacting organisms, the pathogen and the host plant. Here, we analysed *R*. *solani* AG1-IB gene expression in the course of interaction with lettuce. To our knowledge, no comprehensive mRNA-based transcriptome studies have been published previously for this pathogen. Therefore, data analysis by an objective top-down approach has been chosen. Table 1 summarizes the accumulated sequencing and mapping results per interaction zone. A total of 1.3 billion sequence reads (on average approx. 108 million reads for each of three replicates per interaction zone) amounting to 60 Gb sequence information were generated for all transcriptome libraries. Quality filtering resulted in high quality sequence reads that were mapped onto the *R*. *solani* AG1-IB draft genome sequence (Table 1). For Zone 1, the amount of mapped *R*. *solani* reads was 1.67% of all reads, whereas for Zone 2 (75.63%) and Zone 3 (80.56%) reads originating from *R*. *solani* dominated. As expected for Zone 1, the total amount of mapped *R*. *solani* reads was quite low in comparison to the other interaction zones. Therefore, the differentially expressed genes within this zone that are discussed in this paper were manually curated to exclude false positives, the unfiltered data is included in the S2 File.

**Table 1:** Summarized transcriptome sequence read filtering and mapping statistics on the R. solani AG1-IB (isolate 7/3/14) genome sequence.

	Zone 1^1^	Zone 2^1^	Zone 3^1^	Control^1^
**Read counts after filtering and trimming**	348,976,835	296,489,953	283,975,363	335,812,524
**Total mapped reads to the *R*. *solani* AG1-IB genome**	5,821,612	224,235,563	228,798,028	3,866,558
**Percentage of mapped reads [%]**	1.67	75.63	80.56	1.15

^1^The values for the biological replicates are summated within this table, but not for the statistical evaluations unless described so in the methods section. For read counts per specific library, please see the S2 File.

### The most abundant *R*. *solani* transcripts in the three interaction zones

The overall highest expressed genes per interaction zone were analysed by calculation of corresponding RPKM values.

#### The most abundant *R*. *solani* transcripts in Zone 1

Specialized structures required for infection had not been formed and penetration had not taken place in Zone 1. Genes with the highest level of transcript abundance in Zone 1 are listed in Table A in S1 File. RPKM values for all of the expressed genes can be found in S2 File. High transcript abundance of genes encoding transmembrane proteins and proteins of unknown functions was characteristic for this zone.

Within Zone 1, a subset of 21 genes encoding putative transmembrane proteins was highly transcribed. Each of the corresponding gene products comprises one or multiple transmembrane regions and SignalP motifs. The genes of this group have an average nucleotide sequence length of 200 +/- 30 bp. Two sets of three genes each are clustered within the AG1-IB (isolate 7/3/14) genome (*RSOLAG1IB_9721*, *RSOLAG1IB_9722* and *RSOLAG1IB_9723* and *RSOLAG1IB_7751*, *RSOLAG1IB_7750* and *RSOLAG1IB_7749*). Alignments of deduced amino acid sequences revealed relationships of these predicted proteins (Fig A in S1 File). By means of nucleotide BLAST analyses, several similar genes were found within the genomes of different *R*. *solani* isolates that represent the anastomosis groups AG3 (isolate Rhs1AP) and AG8 (isolate WAC10335). The exclusive occurrence of these genes in different *R*. *solani* anastomosis groups suggests that they encode a novel, previously unknown, albeit conserved function within the *R*. *solani* species complex. Further functional analysis of these genes will be necessary to elucidate a putative role of the encoded transmembrane proteins in the early phase of host pathogen interaction.

A transcript similar to the *gEgh16* gene [32] from the biotrophic powdery mildew pathogen *Blumeria graminis*, homologue to *GAS1* in *Magnaporthe grisea* [33], was highly abundant within Zone 1 (*RSOLAG1IB_6054*). The *gEgh16* gene product contains an InterPro DUF 3129 domain (IPR021476) of unknown function. A BLAST search within the *R*. *solani* AG1-IB (isolate 7/3/14) genome revealed three further *gEgh16*-like genes encoding DUF 3129 domains. Knockout mutants of orthologues of the *gEgh16* gene in *M*. *grisea* caused appressoria that were less successful in penetration of rice and barley leaves and therefore were associated with reduced virulence. Xue and co-workers [33] also suggested that expression of these genes may exclusively occur within appressoria. This correlates well with the gEGh16 expression profile observed in this study. Moreover, infection structures were observed by microscopy to occur in Zone 2 on lettuce leaves. It is assumed that *gEgh16*-like genes are involved in appressoria formation of *R*. *solani* AG1-IB on leaf surfaces comparable to the findings for biotrophic pathogens [32,33]. Further transcripts homologues to *gEgh16* and *GAS1* were found within the PHI-base analysis, S1 File.

#### The most abundant *R*. *solani* transcripts in Zone 2

Within Zone 2, *R*. *solani* formed differentiated infection structures and initial necrosis of lettuce cells was evident by an observed decrease in chlorophyll fluorescence. Of note within this zone, a high abundance of transcripts related to ribosome function and general metabolism was observed (Table B in S1 and S2 Files).

More than half of the 20 most highly expressed transcripts in Zone 2 encode ribosomal proteins implying that a high degree of transcript translation is occurring, likely because the organism is most metabolically active in this state. The overall most highly expressed gene in the interaction Zone 2 is *RSOLAG1IB_10881* which encodes a putative ricin-type beta-trefoil lectin domain protein (IPR000172). This protein is also known as *R*. *solani* agglutinin (RSA) and specifically binds a single Gal/GalNAc sugar unit [34]. *RSOLAG1IB_10881* is also the highest expressed gene in Zone 3. In total, 44 ricin-type beta-trefoil lectin domain protein motifs (IPR000172) were found to be encoded within the *R*. *solani* AG1-IB genome. In addition to *RSOLAG1IB_10881*, other genes of this class are also among the most highly transcribed genes within this zone: *RSOLAG1IB_9431*, *RSOLAG1IB_4833*, *RSOLAG1IB_8515* and *RSOLAG1IB_8516*. In contrast, in Zone 1, the abundance of these ricin-type beta trefoil domain protein transcripts is less prominent and none of the corresponding genes occurs within the top 200 of the most highly transcribed genes, with the exception of *RSOLAG1IB_10881*. These results suggest a function of ricin-type beta trefoil domain proteins in later stages of the interaction process and are in accordance with previous findings for *R*. *solani* [35]. The authors Kellens and Peumans hypothesized that *R*. *solani* lectins could function as storage proteins within *R*. *solani* mycelium and sclerotia and therefore accumulate towards the end of each disease cycle when survival structures like sclerotia were formed. Interestingly, *R*. *solani* RSA also has entomotoxic properties and it was hypothesized that it may protect fungal mycelia and sclerotia against insect predation during the survival phase [36, 37].

The *RSOLAG1IB_2430* transcript, which was annotated to putatively encode a benzoquinone reductase, might play a role in regulation or the degradation of aromatic compounds as was suggested for a similar transcript from the basidiomycete fungus *Phanerochaete chrysosporium* [38]

#### The most abundant *R*. *solani* transcripts in Zone 3

Within Zone 3, *R*. *solani* metabolizes the remaining lettuce substrates and prepares for the survival stage to await a new disease cycle. The most abundant transcripts characteristic for this stage of interaction can be found in Table C in S1 and S2 Files.

Besides the high expression of ricin-type beta-trefoil lectin domain proteins *RSOLAG1IB_10881* and *RSOLAG1IB_8516*, the cyanovirin-n homologous *RSOLAG1IB_8704* gene encoding another member of the lectin protein family is also among the 20 most highly expressed genes in Zone 3. Cyanovirin-n was first discovered in the cyanobacterium *Nostoc ellipsosporum* and is of biotechnological interest for its antiviral activity based on efficient mannose glycan binding [39]. It was suggested that these proteins have potential functions within the lifestyle transition of fungi through cell-cell interaction or transmission of metabolic signals [40,41].

Another highly expressed gene encodes the septal pore cap protein (*SPC18*, *RSOLAG1IB_4026*), which is part of the plugging material that closes the perforations within the septal pore cap (SPC) of hyphal cells. This protein is specific for the species *R*. *solani* [42]. The septal pore connects hyphal cells and allows for transport of cytoplasmic fluids as well as organelles between neighboring cells, thus creating continuity of the cytoplasm. In comparison to Zone 1, in Zone 3, the fungus had produced many infection structures and new hyphae. After the complete depletion of lettuce tissue from nutrients within Zone 3, the fungus does not need these differentiated cellular structures any longer. It is hypothesized that *R*. *solani* up-regulates the production of SPC18 to close off the SPCs in order to shut down metabolism in hyphae that are no longer required for nutrient acquisition. This might be important for preservation of energy which the pathogen uses for the formation of other features, such as survival structures at the end of the disease cycle.

In comparison to the top 20 transcripts in Zone 2, less genes can be correlated to ribosomal activity, though some transcripts are present that can be correlated to general metabolism.

### *R*. *solani* differential gene expression between the interaction zones

**Fig 3:**
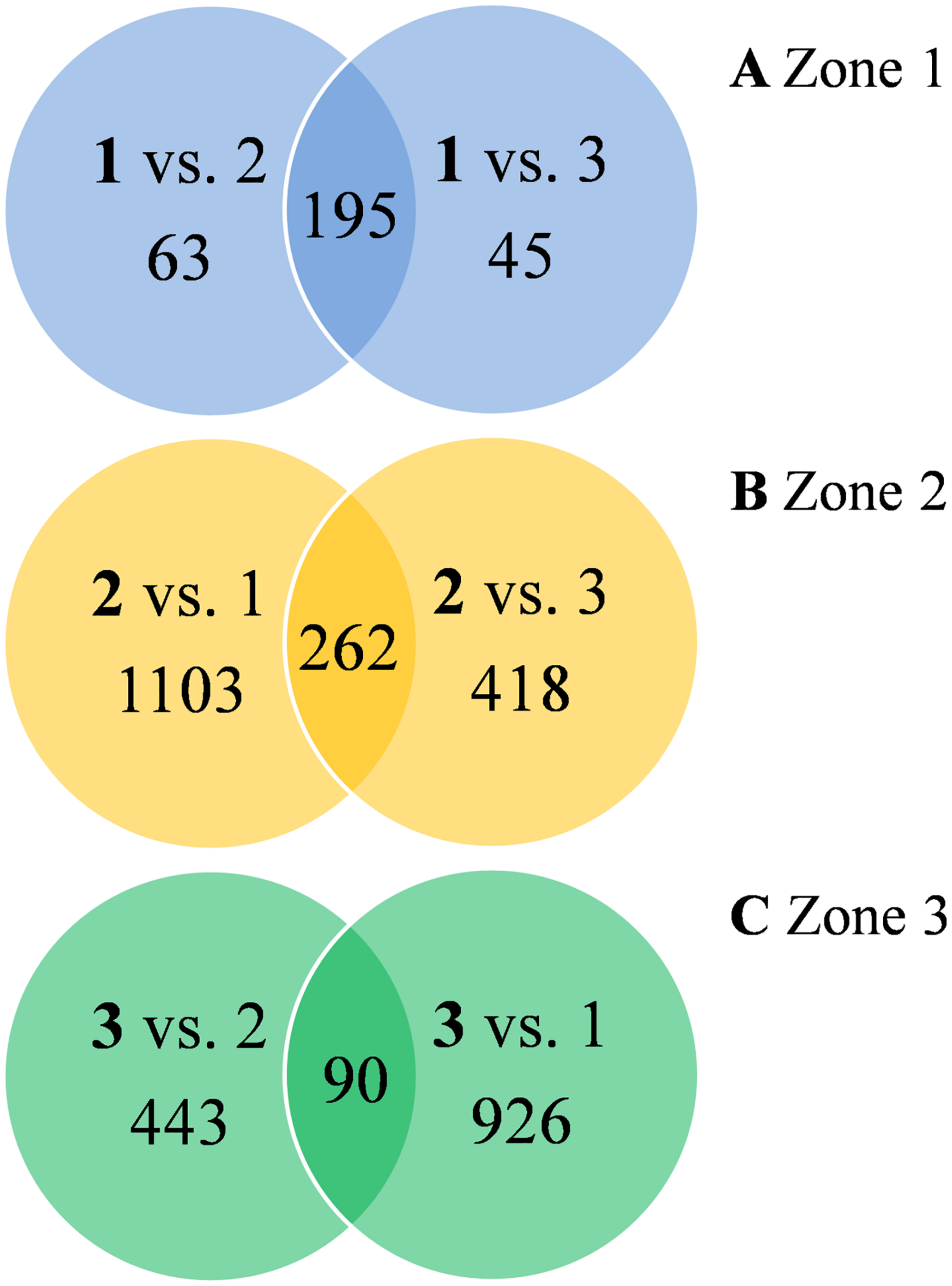
Comparison of differentially expressed genes between interaction zones 1, 2 and 3. Differentially expressed genes in Zone 1 (A), Zone 2 (B), and Zone 3 (C). Values within the intersecting sets represent genes that are more highly expressed when compared to both the other conditions.

In addition to the most highly expressed genes per zone, also differentially expressed genes (DEGs) between the different zones were analysed. The widely established DESeq method for pairwise comparisons was applied for this approach. In total, more than 3500 DEGs were identified between the three interaction zones in pairwise comparisons (Fig 3).

**Fig 4:**
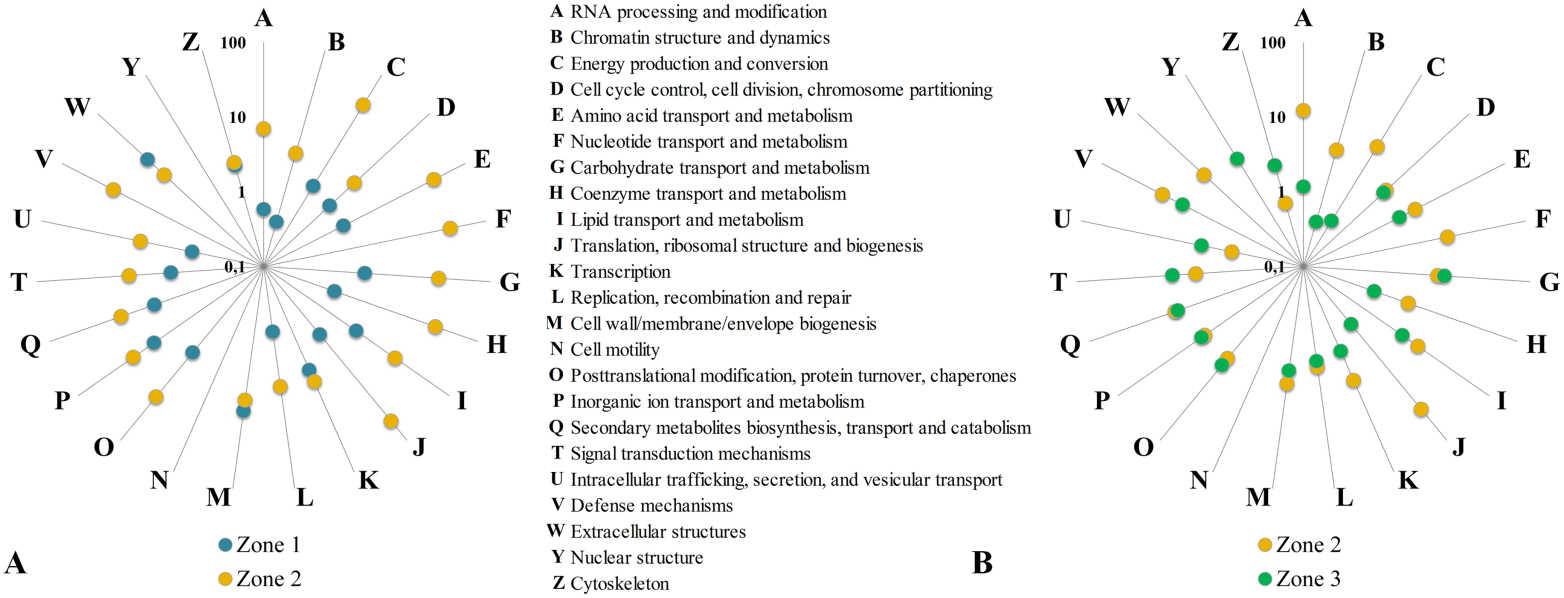
KOG enrichment plot of differentially expressed genes between zones 1, 2 and 3. Results are plotted as a percentage of the total number of *R*. *solani* AG1-IB (isolate 7/3/14) gene products found for each respective functional KOG category in a logarithmic scale. (A) Zone 1 versus Zone 2. (B) Zone 2 versus Zone 3.

The top 10 induced and repressed DEGs from the pairwise comparisons between Zone 1 and Zone 2, and Zone 2 and Zone 3 are listed in Table A and B in S1 File. All of the DEGs were annotated according to the Carbohydrate Active enZyme (CAZy) database (S1 File) and according to KOG (EuKaryotic Orthologous Groups) categories to summarize and depict up-regulation of categorized cellular functions (Fig 4). Furthermore, the top 10 most remarkable DEGs featuring homology to PHI-base pathogenicity or virulence factors of plant pathogens are also listed in Supplementary S1 File.

#### Differentially expressed genes between Zone 1 and Zone 2

With the exception of the KOG category for extracellular structures and cell wall/membrane/envelope biogenesis (category M and W), DEGs representing all other KOG categories were over-represented in Zone 2 compared to Zone 1 (Fig 4A). The most distinctively over-represented function in Zone 2 was the KOG category of translation, ribosomal structure and biogenesis (category J). This result correlates with the observation of a high amount of transcripts encoding ribosomal proteins indicated by the RPKM analyses of the same zone (see above). Similar observations are apparent for several of the other KOG classes that are consistently over-represented within the Zone 2 transcriptome in comparison to the other two zones, albeit to a lesser extent. Thus, it is suggested that the majority of protein synthesis and metabolic activity during the interaction with lettuce takes place within Zone 2 after successful infection of the leaf tissue.

Within Zone 1, the KOG category for extracellular structures was over-represented (category M). Most interestingly, within this category, the genes *RSOLAG1IB_6698* and *RSOLAG1IB_3241* that are annotated to encode putative fasciclin and related adhesion glycoproteins, and the extracellular matrix glycoprotein laminin beta-subunit, respectively, were increased. Proteins containing fasciclin domains have been suggested to play a role in cell adhesion, appressoria turgor set-up and general pathogenesis in several pathogens [43,44]. They may have a function in attachment to the leaf surface in the case of *R*. *solani* AG1-IB.

The most upregulated transcript in Zone 1 in relation to Zone 2 is *RSOLAG1IB_12017* (Table D in S1 File). This transcript encodes a putative protein containing a partial motif related to the *Bacillus thuringiensis* delta-endotoxin CytB (IPR001615). Within our dataset, a second transcript for a similar protein was also found to be up-regulated during this phase of interaction (*RSOLAG1IB_11055*). The various *B*. *thuringiensis* toxins are well known for their insecticidal activity [45]. Currently, it is unclear whether the two corresponding *R*. *solani* putative toxins feature a similar function in the defence against mycophagous insects. It is conceivable that *R*. *solani* produces an insecticidal toxin in this phase of its life cycle, for future protection. However, as mentioned above, many necrotrophs secrete phytotoxic metabolites after entry of host cells for induction of necrosis. Hence, the two *R*. *solani* putative toxins may exert phytotoxic activity in Zone 2 where initial necrosis appears.

Both transcripts *RSOLAG1IB_4413* and *RSOLAG1IB_6086* encode for putative pria proteins that might be involved in the replication of DNA, indicating that in zone 1 the hyphal cells are indeed preparing for proliferation later on in Zone 2.

One of the highest differentially over expressed transcripts in Zone 2 in relation to Zone 1 is encoding a putative gdsl-like lipase acylhydrolase domain containing protein. The only gdsl-like lipase in relation to a phytopathogenic interaction described in literature is *GLIP1* from *Arabidopsis thaliana*, a gene involved in the repression of the necrotrophic fungus *Alternaria brassicicola*, through positive regulation of systemic resistance [46,47]. With the data collected from this experiment alone it is impossible to speculate on the function of this gene in the context of the interaction between *R*. *solani* and *L*. *sativa*.

Additionally, the *RSOLAG1IB_9932* and *RSOLAG1IB_9933* transcripts were significantly up-regulated within Zone 2, although these were not included with the top 10. They both code for putative cytochrome P450 monooxygenase CYP53A, benzoate-para hydroxylases [48]. Such enzymes were frequently found in wood rotting fungi and are involved in the detoxification of plant-derived benzoate derivatives. Furthermore, in *Basidiomycota*, these enzymes were suggested to be involved in the synthesis of veratyl alcohol [49], which may function as redox mediator of peroxidases during the oxidation of lignin.

#### Differentially expressed genes between Zone 2 and Zone 3

Comparison of the transcriptomes of Zone 2 and Zone 3 revealed that the representation of most KOG categories is balanced between these two zones (Fig 4B). In Zone 2, the categories related to biogenesis, metabolism, energy production, chromatin and RNA are clearly enriched (categories A, B, C, F, J and K). Within Zone 3, the category of carbohydrate transport and metabolism is slightly over-represented (category G). The enrichment of these specific KOG categories strengthens the general hypothesis that *R*. *solani* becomes metabolically most active within Zone 2 and starts to prepare for the subsequent formation of survival structures within Zone 3.

The dominance of transcripts classified to represent the KOG category carbohydrate transport and metabolism in Zone 3 is mainly due to the overrepresentation of transcripts coding for proteins containing the conserved protein domain classified as KOG4626. In total, 15 transcripts encoding KOG4626 were identified within this zone *versus* none in Zone 2. KOG4626 represents a conserved domain within O-linked N-acetylglucosamine transferases (OGT). At the biochemical level, OGT catalyses the addition of an N-acetylglucosamine (GlcNAc) monomeric sugar molecule to serine or threonine residues of intracellular proteins. GlcNAc is the monomeric unit of fungal chitin, and OGT may participate in modification and recycling of cell wall material or signalling as it has been suggested for *Candida albicans* [50].

Most prominent within the list of top scoring differentially expressed genes between Zone 2 and 3 is the presence of several genes whose products can be coupled directly or indirectly to the degradation of lignin (Table E in S1 File). *RSOLAG1IB_10268*, *RSOLAG1IB_8324*, *RSOLAG1IB_1987* and *RSOLAG1IB_4082* are putatively annotated as pectin lyase, carbohydrate esterase family protein 4, pectate lyase and a pyranose 2-oxidase. The latter is known from *Peniophora sp*., to be involved in lignin degradation [51]. These results correlate with our hypothesis that *R*. *solani* will perform the bulk of complex substrate metabolism between the Zones 2 and 3. More genes related to degradation are described within the CAZy chapter in S1 File.

Also expressed within Zone 2, but most abundant in Zone 3 was a group of genes that can putatively be coupled to programmed cell death (PCD). Among these are several transcripts encoding putative meta-caspases that play a role in the initiation of PCD in plant and fungal species. For *M*. *grisea* it was shown that PCD by means of autophagy is of importance during infection of *Oryza sativa* [52]. Yoshimoto et al. [53] suggested that the process of pexophagy specifically, may play a role in the recycling of acetyl-CoA and organelles for cell wall integrity and melanin biosynthesis in *M*. *grisea*, thereby optimizing the energetic requirements for infection. Comparing Zone 1 with Zone 2, already four transcripts encoding meta-caspases are significantly increased, while comparing Zone 2 with Zone 3 six further putative meta-caspase genes appeared to be significantly up-regulated. This observation of increased transcript abundance with disease progression strongly suggests that apoptosis specifically plays a role at the end of the *R*. *solani* disease cycle. A number of other genes predicted to be involved in protection of cells from PCD were found to be up-regulated in Zone 2. Ceramidases facilitate the cleavage of fatty acids from the membrane compound ceramide. The product of this reaction is sphingosine which in turn can be phosphorylated by sphingosine kinase. The phosphorylated product sphingosine-1-phosphate (S1P) can protect cells from apoptosis. Interestingly, a putative ceramidase gene (*RSOLAG1IB_5781*) was up-regulated within Zone 2, while the sphingosine kinase gene (*RSOLAG1IB_964*) was not regulated in any of the tested interaction zones.

## Concluding remarks

The results of the current study demonstrate that the necrotrophic pathogen *R*. *solani* AG1-IB shows specific gene expression patterns during the course of interaction with its host plant lettuce. Significantly higher expressed genes in Zone 1, Zone 2 and Zone 3 are related to distinct functions of pathogenesis such as formation of infection structures, suppression of the plant defense response, induction of necrosis or nutrient acquisition. Several of the identified and discussed genes appeared to have predicted roles in the *R*. *solani* life cycle, and therefore should represent key targets for future research. Unfortunately, it is not possible to confirm predicted pathogenic functions by genetic engineering methods since so far, no transformation protocol has been established for *R*. *solani* AG1-IB. Further work is required to overcome this limitation which may be possible since transformation methods have been published for other *R*. *solani* AGs [54,55]. However, based on results obtained in the current study, possible targets for antagonistic measures already can be suggested such as blocking of initial signaling cascades and/or fungal differentiation processes.

Finally, since the central transcriptome sequencing approach described in this study was conducted in a dual manner to address fungal as well as lettuce transcripts, the next step in the analysis certainly will be to investigate the transcriptional response of lettuce towards interaction with *R*. *solani* AG1-IB. A prerequisite for this approach is the availability of a suitable lettuce reference genome sequence, preferably for the cultivar Tizian used in corresponding experiments.

Findings obtained in this study led to a deeper understanding of the pathogenic interaction of *R*. *solani* AG1-IB (isolate 7/3/14) with its host plant lettuce (*L*. *sativa*) and represent a resource that can be used for the development of rational strategies with the objective of plant disease control.

## Supporting information

S1 File Tables and extra chapters. (DOCX)

S2 File Raw read counts DESeq and RPKM values. (XLSX)
