# Supplementary material for: The Rhizoctonia solani AG1-IB (isolate 7/3/14) transcriptome during interaction with the host plant lettuce (Lactuca sativa L.)
Source: PLoS One. 2017 May 9;12(5):e0177278. doi: 10.1371/journal.pone.0177278 (PMC5423683; doi:10.1371/journal.pone.0177278)
Supplement: S1 File — (DOCX) [file pone.0177278.s001.docx]

**The *Rhizoctonia solani* AG1-IB (isolate 7/3/14) Transcriptome during interaction with the Host Plant *Lactuca sativa –* S1 File**

Bart Verwaaijen^1,2^, Daniel Wibberg^1^, Magdalena Kröber^1^, Anika Winkler^1^, Rita Zrenner^2^, Hanna Bednarz^1^, Karsten Niehaus^1^, Rita Grosch^2^, Alfred Pühler^1^, Andreas Schlüter^1*^

^1^Center for Biotechnology, Bielefeld University, Bielefeld, Germany

^2^Leibniz-Institute of Vegetable and Ornamental Crops (IGZ), Großbeeren, Germany

*Corresponding author

E-mail: aschluet@cebitec.uni-bielefeld.de (AS)

# S1 File. Tables and extra chapters.

**Table of contents**

- Fig A. Sequence alignment of 21 putative transmembrane proteins whose corresponding genes were overexpressed in the early zone of interaction (zone 1).
- Tables A, B and C. Most abundant *R. solani* transcripts per interaction zone
- Supplementary Tables D and E. Pairwise DESeq analysis of differential transcription between the interaction zones
- Chapter A. Results and discussion of Pathogen Host Interaction database (PHI-base) enrichment of DEGs
- Tables F, G, H and I. PHI-base analysis of the respective DEGs
- Chapter B. Results and discussion of Carbohydrate-Active enZYmes
- Fig B, C, D and E. Heat maps of the respective putative CAZy families
- References


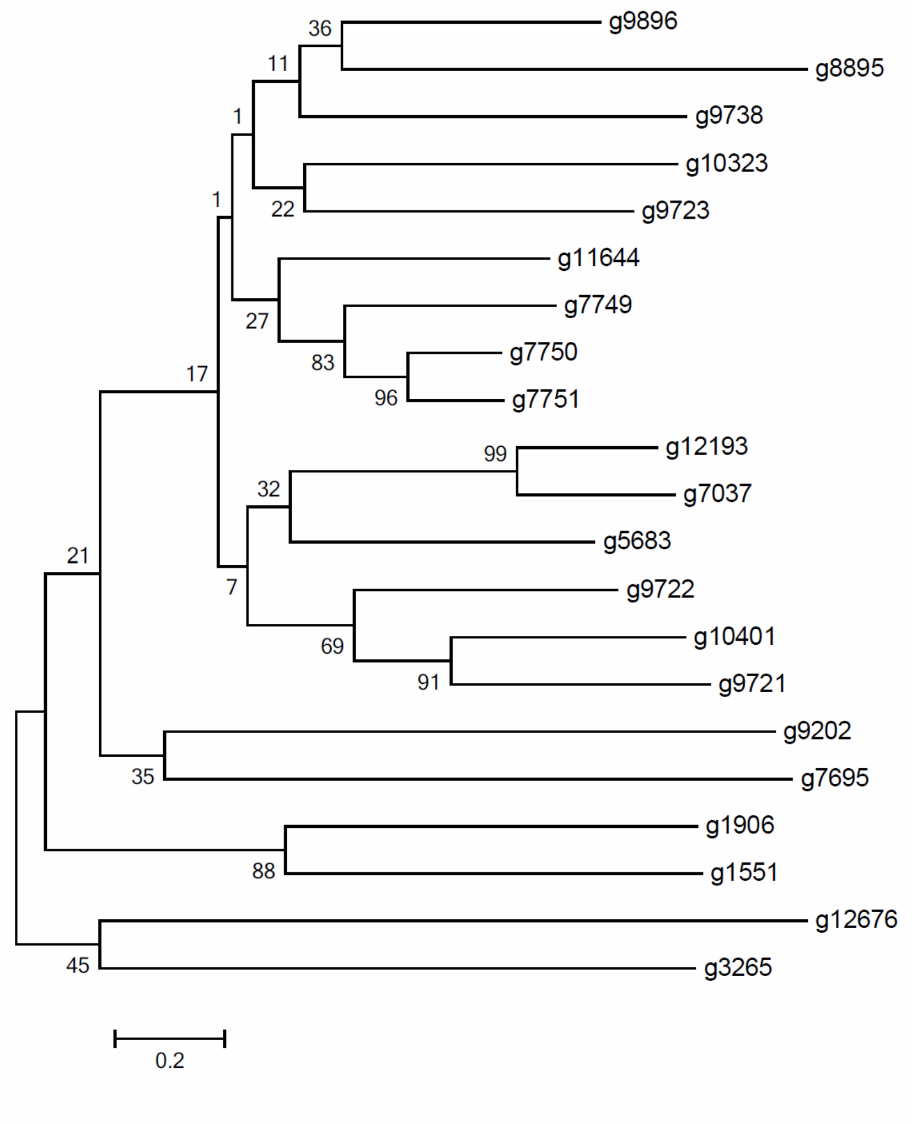


**Fig A. Sequence alignment of 21 putative transmembrane proteins whose corresponding genes were overexpressed in the early zone of interaction (zone 1).**

| **Table A. Most abundant *R. solani* transcripts in interaction zone 1** | | | |
| --- | --- | --- | --- |
|  | Genome locus | RPKM value^1^ | Putative function^2^ |
| 1 | RSOLAG1IB_8895 | 10941 | transmembrane protein |
| 2 | RSOLAG1IB_7751 | 10154 | transmembrane protein |
| 3 | RSOLAG1IB_7750 | 7670 | transmembrane protein |
| 4 | RSOLAG1IB_6054 | 6395 | gegh 16 partial |
| 5 | RSOLAG1IB_4316 | 6186 | Mannoprotein |
| 6 | RSOLAG1IB_9896 | 4956 | transmembrane protein |
| 7 | RSOLAG1IB_8160 | 4715 | hmp1-mismatch base pair and cruciform dna recognition |
| 8 | RSOLAG1IB_12193 | 4711 | transmembrane protein |
| 9 | RSOLAG1IB_7037 | 4240 | transmembrane protein |
| 10 | RSOLAG1IB_10401 | 3772 | transmembrane protein |
| 11 | RSOLAG1IB_3526 | 3312 | hypothetical protein BN14_00464 |
| 12 | RSOLAG1IB_2220 | 3253 | histone h2a |
| 13 | RSOLAG1IB_1478 | 2831 | symbiosis-like protein |
| 14 | RSOLAG1IB_7665 | 2794 | histone-fold-containing protein |
| 15 | RSOLAG1IB_12399 | 2690 | histone h2b |
| 16 | RSOLAG1IB_4413 | 2681 | pria protein |
| 17 | RSOLAG1IB_5946 | 2678 | alcohol dehydrogenase zinc-binding domain protein |
| 18 | RSOLAG1IB_9662 | 2644 | metalloprotease protein |
| 19 | RSOLAG1IB_11644 | 2629 | transmembrane protein |
| 20 | RSOLAG1IB_1551 | 2516 | transmembrane protein |
| ^1^RPKM values calculated over the summated read counts for three biological replicates. Values were normalized according to library sizes and gene lengths. | | | |
| ^2^Putative functions based on BLAST2GO protein BLAST. | | | |

| **Table B. Most abundant *R. solani* transcripts in interaction zone 2** | | | |
| --- | --- | --- | --- |
|  | Genome locus | RPKM value^1^ | Putative function^2^ |
| 1 | RSOLAG1IB_10881 | 17035 | ricin-type beta-trefoil lectin domain protein |
| 2 | RSOLAG1IB_2841 | 7801 | translation elongation factor 1a |
| 3 | RSOLAG1IB_2455 | 7510 | ribosomal protein s25 |
| 4 | RSOLAG1IB_10489 | 7374 | 40s ribosomal protein s26 |
| 5 | RSOLAG1IB_10676 | 6675 | ubiquitin-domain-containing protein |
| 6 | RSOLAG1IB_2863 | 6523 | 40s ribosomal protein s3ae |
| 7 | RSOLAG1IB_6188 | 6482 | 60s acidic ribosomal protein p1 |
| 8 | RSOLAG1IB_4551 | 5993 | 60s ribosomal protein l37 |
| 9 | RSOLAG1IB_8871 | 5800 | nad-dependent formate dehydrogenase |
| 10 | RSOLAG1IB_4084 | 5776 | ribosomal protein s12 s23 |
| 11 | RSOLAG1IB_4490 | 5537 | 60s acidic ribosomal protein p2 |
| 12 | RSOLAG1IB_2430 | 5324 | benzoquinone reductase |
| 13 | RSOLAG1IB_12619 | 5298 | 60s ribosomal protein l24 |
| 14 | RSOLAG1IB_7234 | 5153 | thioredoxin-dependent peroxidase |
| 15 | RSOLAG1IB_2217 | 5123 | 60s ribosomal protein l27a |
| 16 | RSOLAG1IB_2237 | 5122 | eukaryotic adp atp carrier |
| 17 | RSOLAG1IB_991 | 5015 | 40s ribosomal protein s21 |
| 18 | RSOLAG1IB_3726 | 5011 | duf1960-domain-containing protein |
| 19 | RSOLAG1IB_10924 | 4948 | ubiquitin-60s ribosomal protein l40 fusion protein |
| 20 | RSOLAG1IB_5497 | 4925 | 60s ribosomal protein l9 |
| ^1^RPKM values calculated over the summated read counts for three biological replicates. Values were normalized according to library sizes and gene lengths. | | | |
| ^2^Putative functions based on BLAST2GO protein BLAST. | | | |

| **Table C. Most abundant *R. solani* transcripts in interaction zone 3** | | | |
| --- | --- | --- | --- |
|  | Genome loci | RPKM value^1^ | Putative function^2^ |
| 1 | RSOLAG1IB_10881 | 28270 | ricin-type beta-trefoil lectin domain protein |
| 2 | RSOLAG1IB_2430 | 12724 | benzoquinone reductase |
| 3 | RSOLAG1IB_2841 | 6810 | translation elongation factor 1a |
| 4 | RSOLAG1IB_7619 | 6767 | glyceraldehyde-3-phosphate dehydrogenase |
| 5 | RSOLAG1IB_8516 | 6389 | ricin-type beta-trefoil lectin domain protein |
| 6 | RSOLAG1IB_6654 | 5322 | No BLAST hit |
| 7 | RSOLAG1IB_12715 | 5028 | No BLAST hit |
| 8 | RSOLAG1IB_8515 | 5005 | carbohydrate-binding module family 13 protein |
| 9 | RSOLAG1IB_2237 | 4932 | eukaryotic adp atp carrier |
| 10 | RSOLAG1IB_4026 | 4609 | septal pore cap protein spc18 |
| 11 | RSOLAG1IB_214 | 4585 | peptidyl-prolyl cis-trans isomerase |
| 12 | RSOLAG1IB_11672 | 4459 | alcohol dehydrogenase zinc-binding domain protein |
| 13 | RSOLAG1IB_3726 | 4192 | duf1960-domain-containing protein |
| 14 | RSOLAG1IB_10845 | 3874 | Calmodulin |
| 15 | RSOLAG1IB_10489 | 3859 | 40s ribosomal protein s26 |
| 16 | RSOLAG1IB_3758 | 3722 | nucleoside diphosphate kinase |
| 17 | RSOLAG1IB_7612 | 3639 | dna-binding helix turn-helix protein |
| 18 | RSOLAG1IB_8704 | 3445 | cyanovirin-n family protein |
| 19 | RSOLAG1IB_2863 | 3430 | 40s ribosomal protein s3ae |
| 20 | RSOLAG1IB_10676 | 3313 | ubiquitin-domain-containing protein |
| ^1^RPKM values calculated over the summated read counts for three biological replicates. Values were normalized according to library sizes and gene lengths. | | | |
| ^2^Putative functions based on BLAST2GO protein BLAST. | | | |

| **Table D. DESeq analysis of differential transcription in zone 1 *vs.* zone 2** | | | | | |
| --- | --- | --- | --- | --- | --- |
|  | Genome locus | log_2_ Fold change^1^ | Padj^1^ | Ø RPKM^2^ | Putative function^3^ |
| 1 | RSOLAG1IB_12017 | -10.8 ↓ | 2.45E-02 | 148,1 | thuringiensis toxin domain protein |
| 2 | RSOLAG1IB_4413 | -10.1 ↓ | 3.19E-09 | 894,3 | pria protein |
| 3 | RSOLAG1IB_673 | -9.9 ↓ | 1.16E-02 | 193,9 | transmembrane protein |
| 4 | RSOLAG1IB_10850 | -9.8 ↓ | 4.09E-13 | 7,7 | elongator complex protein 2 |
| 5 | RSOLAG1IB_6086 | -9.6 ↓ | 1.13E-10 | 455,0 | pria protein |
| 6 | RSOLAG1IB_3391 | -9.6 ↓ | 3.73E-04 | 92,7 | hypothetical protein RSOLAG1IB_03391 |
| 7 | RSOLAG1IB_10406 | -9.3 ↓ | 7.10E-05 | 17,8 | transmembrane protein |
| 8 | RSOLAG1IB_8951 | -9.3 ↓ | 2.92E-03 | 4,7 | amp-binding enzyme |
| 9 | RSOLAG1IB_8105 | -9.2 ↓ | 4.21E-03 | 15,6 | hypothetical protein RSOLAG1IB_08101 |
| 10 | RSOLAG1IB_7003 | -9.0 ↓ | 7.96E-03 | 48,5 | cytochrome p450 |
| 11 | RSOLAG1IB_1609 | ∞ ↑ | 4.91E-04 | 20,1 | carbohydrate esterase family 16 protein |
| 12 | RSOLAG1IB_8524 | ∞ ↑ | 9.90E-03 | 12,8 | plc-like phosphodiesterase |
| 13 | RSOLAG1IB_7810 | ∞ ↑ | 1.01E-02 | 19,9 | alpha beta-hydrolase |
| 14 | RSOLAG1IB_6095 | ∞ ↑ | 1.44E-02 | 15,7 | transposon ty3-g gag-pol polyprotein |
| 15 | RSOLAG1IB_12107 | ∞ ↑ | 3.77E-02 | 25,9 | tyrosine kinase catalytic domain protein |
| 16 | RSOLAG1IB_4760 | ∞ ↑ | 4.17E-02 | 19,1 | alpha beta hydrolase family protein |
| 17 | RSOLAG1IB_6942 | ∞ ↑ | 4.24E-02 | 9,2 | serine protease |
| 18 | RSOLAG1IB_6845 | ∞ ↑ | 1.51E-04 | 13,7 | gdsl-like lipase acylhydrolase domain protein |
| 19 | RSOLAG1IB_8532 | ∞ ↑ | 1.45E-02 | 10,1 | s-adenosyl-l-methionine-dependent methyltransferase |
| 20 | RSOLAG1IB_3585 | ∞ ↑ | 4.25E-02 | 22,7 | oxalate decarboxylase |
| ^1^Values calculated with the DESeq R package as it is implemented in the ReadXplorer software. | | | | | |
| ^2^Average RPKM values during the experiment as calculated over all interaction zones. | | | | | |
| ^3^Putative functions based on BLAST2GO protein BLAST analyses. | | | | | |
|  | | | | | |

| **Table E. DESeq analysis of differential transcription in zone 2 *vs.* zone 3** | | | | | | | | | | | |
| --- | --- | --- | --- | --- | --- | --- | --- | --- | --- | --- | --- |
|  | Feature | log_2_ Fold change^1^ | | Padj^1^ | | Ø RPKM^2^ | | Putative function^3^ | |  |  |
| 1 | RSOLAG1IB_10475 | | -4.9 ↓ | | 1.16E-41 | | 88,8 | | AA9 protein (former GH61) | |  |
| 2 | RSOLAG1IB_10268 | | -4.7 ↓ | | 3.55E-02 | | 0,1 | | pectin lyase | |  |
| 3 | RSOLAG1IB_10509 | | -4.3 ↓ | | 1.27E-02 | | 0,8 | | general substrate transporter | |  |
| 4 | RSOLAG1IB_11357 | | -4.0 ↓ | | 7.54E-07 | | 4,2 | | transmembrane partial | |  |
| 5 | RSOLAG1IB_10438 | | -3.8 ↓ | | 1.80E-05 | | 63,2 | | hypothetical protein BN14_11558 | |  |
| 6 | RSOLAG1IB_9120 | | -3.7 ↓ | | 1.75E-05 | | 4,9 | | tyrosinase tyrosinase: common central domain protein | |  |
| 7 | RSOLAG1IB_9822 | | -3.4 ↓ | | 2.59E-02 | | 0,4 | | protocatechuate -dioxygenase beta partial | |  |
| 8 | RSOLAG1IB_5861 | | -3.3 ↓ | | 3.29E-09 | | 3,1 | | zinc-type alcohol dehydrogenase-like protein | |  |
| 9 | RSOLAG1IB_8324 | | -3.3 ↓ | | 1.09E-04 | | 138,3 | | carbohydrate esterase family 4 protein | |  |
| 10 | RSOLAG1IB_1987 | | -3.2 ↓ | | 2.66E-02 | | 38,0 | | pectate lyase | |  |
| 11 | RSOLAG1IB_10412 | | 3.3 ↑ | | 3.34E-19 | | 20,8 | | No BLAST hit | |  |
| 12 | RSOLAG1IB_2012 | | 3.4 ↑ | | 4.81E-02 | | 0,5 | | aromatic di-alanine and tpr containing protein | |  |
| 13 | RSOLAG1IB_5357 | | 3.4 ↑ | | 4.10E-02 | | 0,3 | | ibr domain protein | |  |
| 14 | RSOLAG1IB_1636 | | 3.5 ↑ | | 1.50E-02 | | 0,5 | | ice-like protease p20 domain protein | |  |
| 15 | RSOLAG1IB_12047 | | 3.6 ↑ | | 6.61E-03 | | 0,1 | | 50s ribosome-binding gtpase | |  |
| 16 | RSOLAG1IB_12786 | | 3.8 ↑ | | 1.97E-03 | | 28,0 | | high-affinity nicotinic acid transporter | |  |
| 17 | RSOLAG1IB_4082 | | 3.8 ↑ | | 2.84E-02 | | 21,0 | | pyranose 2-oxidase | |  |
| 18 | RSOLAG1IB_10002 | | 4.1 ↑ | | 3.56E-02 | | 0,1 | | 50s ribosome-binding gtpase | |  |
| 19 | RSOLAG1IB_12000 | | 4.4 ↑ | | 3.34E-03 | | 0,2 | | transmembrane partial | |  |
| 20 | RSOLAG1IB_7615 | | 4.5 ↑ | | 1.03E-02 | | 37,5 | | major facilitator superfamily transporter | |  |
| ^1^Values calculated with the DESeq R package as it is implemented in the ReadXplorer software. | | | | | | | | | | | |
| ^2^Average RPKM values during the experiment as calculated over all interaction zones. | | | | | | | | | | | |
| ^3^Putative functions based on BLAST2GO protein BLAST analyses. | | | | | | | | | | | |

# Results and discussion of Pathogen Host Interaction database (PHI-base) enrichment of DEGs

The products of three genes were found to correspond to FET3 ferroxidase, two of these being over-expressed within zone 1 and the third being over-expressed within zone 3. A more precise analysis of these genes applying the Laccase Engineering Database (LccED) revealed that the encoded proteins are closely related to SF1A family laccases representing phenol oxidases that potentially have functions in detoxification and degradation of lignin [1].

Deduced products of transcripts RSOLAG1IB_910 and RSOLAG1IB_1714 share homology with GLO1 glyoxal oxidase that releases hydrogen peroxide [2] and is required for full pathogenicity of *U. maydis*. Several functions have been proposed for this enzyme such as cell wall maturation, regulation of ROS, lignin degradation or signalling.

Several genes encoding subtilisin-like serine proteases sharing homology with *M. oryzae* SPM1 were upregulated in zone 2 compared to zone 1. These genes also display a similar degree of similarity to *Metarhizium anisopliae* PR1 cuticle-degrading protease. Analysis of their genomic loci revealed that at least two sets of paralogous genes exist within the *R. solani* AG1-IB (isolate 7/3/14) genome. One set comprises three genes (RSOLAG1IB_10664, RSOLAG1IB_10665, RSOLAG1IB_1066) whereas the second set features five homologous genes (RSOLAG1IB_7810 to RSOLAG1IB_7814), respectively.

RSOLAG1IB_ expressed within zone 1 shares homology with the Ras protein MoTea4 which is essential in polarized growth and development of appressoria in *M.* *oryzae* [3]*.* The transcript of RSOLAG1IB_4886 was also expressed in zone 1 and encodes a putative Ypk1/Gad8/Aga1 AGC Ser/Thr kinase that in *U. maydis* is essential for appressorium formation and actin cytoskeleton maintenance [4]. These two transcripts suggest the presence of a tight regulation for appressorium formation between zone 1 and 2.

The protein of transcript RSOLAG1IB_8343 contains a partial Gti/Pac2 motif that has potential functions in sexual development, pathogenesis and regulation of gluconate uptake under glucose starvation conditions.

Within zone 2, the protein of RSOLAG1IB_8359 is homologous to the *U. maydis* chimeric bifunctional spermidine synthase and saccharopine dehydrogenase which is essential for mycelium morphogenesis and *in planta* survival of this pathogen [5]. The gene RSOLAG1IB_8356 encodes for a putative lysine-ketoglutarate reductase/saccharopine dehydrogenase that also is up-regulated within zone 2.

Two heat shock protein (hsp70) SSB transcripts (RSOLAG1IB_4239 and RSOLAG1IB_877) were found to be up-regulated within zone 2. Corresponding ribosome associated proteins are involved in protein folding and knockout of the LHS1 hsp70 ER chaperone in *M. oryzae* severely reduced pathogenicity [6].

The RSOLAG1IB_1407 transcript encodes a homologue of the *M. oryzae* trehalose-6-phosphate synthase (Tps1), a protein suggested to regulate post-penetration genetic reprogramming and functions in appressoria turgor generation [7,8]. Interestingly, the product of the enzymatic reaction, the alpha-linked disaccharide trehalose, is known as an inducer of plant defense mechanisms and a virulence factor in both fungal and bacterial phytopathogens. Aliferis & Jabaji [9] reported on an elevation of trehalose concentrations in *Solanum tuberosum* sprouts infected with *R. solani* AG3.

The gene RSOLAG1IB_4365 encoding a putative multi sensor signal transduction histidine kinase could not be annotated specifically. It is likely that this protein is involved in signal perception and/or transduction as it appears to be a member of the two-component sensor kinases containing both a CheY-like (IPR011006) and PAS (IPR000014) domain.

The transcripts of RSOLAG1IB_6281, RSOLAG1IB_8826 and RSOLAG1IB_8394 all encode for putative sugar transporters and most likely function in sequestration of extracellular carbon.

In zone 3, several transcripts (RSOLAG1IB_6135, RSOLAG1IB_6145 and RSOLAG1IB_12659) representing WD40 motif proteins were over-expressed. WD40 motifs for example are present in the Tup1 general transcription repressor from *U. maydis* or the HET-E vegetative incompatibility proteins from *Podospora anserine* [10,11]. Moreover, WD40 motifs play a role in PCD as activators of caspases. In total, 24 genes coding for WD40 repeat containing proteins were up-regulated within zone 3.

Furukawa & Syono [12] discovered that *R.* *solani* increases its production of auxin when cultivated in medium substituted with rice culture filtrate. The product of RSOLAG1IB_5930 is a homologue of the *Colletotrichum gloeosporioides* CgOPT1 oligopeptide transporter involved in the reaction towards auxin of this fungus [13]. In addition, two further genes (RSOLAG1IB_2537 and RSOLAG1IB_9250) for potential auxin efflux carriers were identified; RSOLAG1IB_9250 was significantly upregulated within zone 3.

Finally, the deduced protein sequences of RSOLAG1IB_7338 and RSOLAG1IB_10292 represent hypothetical proteins featuring homology to non-ribosomal peptide synthetases possessing a wide variety of functions, for example *Alternaria brassicicola* TmpL orthologues were suggested to contribute to ROS homeostasis in both plant and animal pathogenic fungi [14].

| **Table F. PHI-base analysis of DEGs upregulated in zone 1 *vs*. 2** | | | | | | | |  |
| --- | --- | --- | --- | --- | --- | --- | --- | --- |
|  | Genome | Gene name^1^ | PHI^1^ | e-value^1^ | Functional description^2^ | Log_2_ | Average | |
|  | Locus |  |  |  |  | Fold change^3^ | RPKM^4^ | |
| 1 | RSOLAG1IB_6123 | FET3-1 | 2920 | 2E-38 | Functional ferroxidase | 7,2 | 5,3 | |
| 2 | RSOLAG1IB_8722 | FET3-2 | 2921 | 1E-60 | Functional ferroxidase | 7,0 | 37,5 | |
| 3 | RSOLAG1IB_1906 | GAS1 | 256 | 4E-43 | Appressorial penetration | 6,7 | 627,5 | |
| 4 | RSOLAG1IB_5157 | GAS1 | 256 | 2E-27 | Appressorial penetration | 6,6 | 630,9 | |
| 5 | RSOLAG1IB_8106 | FGSG_03146 | 1221 | 1E-59 | protein kinase | 5,4 | 44,5 | |
| 6 | RSOLAG1IB_910 | GLO1 | 352 | 6E-113 | Glyoxal oxidase | 5,3 | 164,2 | |
| 7 | RSOLAG1IB_10664 | SPM1 | 2117 | 2E-49 | protease activity | 5,1 | 37,8 | |
| 8 | RSOLAG1IB_7344 | CTB5 | 1046 | 1E-42 | Oxidoreductase | 4,7 | 19,6 | |
| 9 | RSOLAG1IB_117 | MGG_04985 | 882 | 3E-84 | succinate dehydrogenase subunit | 4,5 | 31,4 | |
| 10 | RSOLAG1IB_1719 | tmpL | 2296 | 2E-41 | Intracellular Redox Homeostasis | 4,5 | 56,4 | |
| 11 | RSOLAG1IB_8668 | Gas1 | 1071 | 2E-21 | encoding an endoplasmic reticulum glucosidase 2 | 4,0 | 8,0 | |
| 12 | RSOLAG1IB_10529 | CBL1 | 443 | 2E-45 | Cystathionine beta-lyase | 3,9 | 18,5 | |
| 13 | RSOLAG1IB_6122 | FET3-1 | 2920 | 8E-48 | Functional ferroxidase | 3,8 | 11,4 | |
| 14 | RSOLAG1IB_8488 | Bcchs3a | 2359 | 3E-168 | Chitin Synthase | 3,6 | 48,6 | |
| 15 | RSOLAG1IB_6270 | AOX1 | 199 | 2E-141 | Alcohol oxidase | 3,4 | 226,9 | |
| 16 | RSOLAG1IB_4555 | KIN2 | 465 | 1E-58 | Kinesin | 2,9 | 15,2 | |
| 17 | RSOLAG1IB_4886 | Aga1 | 2246 | 0.0 | AGC Ser/Thr kinase | 2,8 | 101,1 | |
| 18 | RSOLAG1IB_8343 | Fgp1 | 2399 | 2E-36 | Toxin Synthesis. pathogenicity and Reproduction | 2,7 | 176,5 | |
| 19 | RSOLAG1IB_1714 | GLO1 | 352 | 1E-152 | Glyoxal oxidase | 2,7 | 105,5 | |
| 20 | RSOLAG1IB_3677 | MoTea4 | 2180 | 1E-35 | Regulate polarized growth and morphogenesis | 2,6 | 14,6 | |
| ^1^Gene name, PHI-base identifier and e-values as obtained from a protein BLAST against the PHI-base database. | | | | | | | |  |
| ^2^Putative functions based on BLAST2GO protein BLAST. | | | | | | | |  |
| ^3^Values calculated with the DESeq R package as it is implemented in the ReadXplorer software. | | | | | | | |  |
| ^4^Average RPKM during the experiment calculated over all interaction zones. | | | | | | | |  |

| **Table G. PHI-base analysis of DEGs upregulated in zone 2 *vs*. 1** | | | | | | | |
| --- | --- | --- | --- | --- | --- | --- | --- |
|  | Genome | Gene name^1^ | PHI1 | e-value^1^ | Functional description^2^ | Log_2_ | Average |
|  | Locus |  |  |  |  | Fold change^3^ | RPKM^4^ |
| 1 | RSOLAG1IB_7810 | SPM1 | 2117 | 1E-48 | Protease activity | ∞ | 19,9 |
| 2 | RSOLAG1IB_3271 | CTB4 | 2329 | 6E-47 | Encodes a putative membrane transporter | 5,5 | 9,0 |
| 3 | RSOLAG1IB_8162 | SPM1 | 2117 | 1E-52 | Protease activity | 3,6 | 267,6 |
| 4 | RSOLAG1IB_9845 | PELB | 222 | 9E-76 | Pectate lyase | 3,6 | 14,7 |
| 5 | RSOLAG1IB_7812 | SPM1 | 2117 | 4E-52 | Protease activity | 3,6 | 303,7 |
| 6 | RSOLAG1IB_7895 | MGG_10510 | 811 | 4E-27 | Hypothetical protein | 3,5 | 102,1 |
| 7 | RSOLAG1IB_10258 | pnl1 | 3226 | 8E-69 | pectin lyase | 3,4 | 34,0 |
| 8 | RSOLAG1IB_11677 | RED1 | 2839 | 1E-25 | Reductase | 3,4 | 87,2 |
| 9 | RSOLAG1IB_10666 | SPM1 | 2117 | 1E-55 | Protease activity | 3,3 | 627,3 |
| 10 | RSOLAG1IB_2528 | - | 2209 | 5E-77 | Endo-beta-1,4 xylanase | 3,2 | 120,8 |
| 11 | RSOLAG1IB_4264 | BRM2 | 2844 | 2E-26 | 1,3,8-trihydroxynaphthalene reductase | 3,2 | 224,1 |
| 12 | RSOLAG1IB_877 | LHS1 | 2058 | 1E-29 | Endoplasmic Reticulum Chaperone | 3,2 | 913,9 |
| 13 | RSOLAG1IB_7140 | - | 2393 | 3E-62 | Related to O-methylsterigmatocystin oxidoreductase | 3,1 | 136,5 |
| 14 | RSOLAG1IB_2644 | BUF1 | 2022 | 1E-31 | No description | 3,1 | 150,2 |
| 15 | RSOLAG1IB_986 | ABC3 | 2042 | 3E-34 | multidrug resistance; protection from oxidative stress | 3,1 | 8,8 |
| 16 | RSOLAG1IB_7813 | SPM1 | 2117 | 5E-50 | Protease activity | 3,0 | 79,2 |
| 17 | RSOLAG1IB_7814 | SPM1 | 2117 | 6E-54 | Protease activity | 3,0 | 69,5 |
| 18 | RSOLAG1IB_1407 | TPS1 | 2172 | 1E-86 | Regulation of nitrate metabolism | 2,9 | 36,6 |
| 19 | RSOLAG1IB_8359 | Spe-Sdh | 2239 | 0.0 | encodes spermidine synthase/saccharopine dehydrogenase | 2,9 | 144,4 |
| 20 | RSOLAG1IB_3678 | ADE2 | 14 | 0.0 | Phosphoribosylaminoimidazole carboxylase | 2,9 | 37,8 |
| ^1^Gene name, PHI-base identifier and e-values as obtained from a protein BLAST against the PHI-base database. | | | | | | | |
| ^2^Putative functions based on BLAST2GO protein BLAST. | | | | | | | |
| ^3^Values calculated with the DESeq R package as it is implemented in the ReadXplorer software. | | | | | | | |
| ^4^Average RPKM during the experiment calculated over all interaction zones. | | | | | | | |

| **Table H. PHI-base analysis of DEGs upregulated in zone 2 *vs*. 3** | | | | | | |  |  |
| --- | --- | --- | --- | --- | --- | --- | --- | --- |
|  | Genome | Gene name^1^ | PHI^1^ | e-value^1^ | Function description^2^ | Log_2_ | | Average |
|  | Locus |  |  |  |  | Fold change^3^ | | RPKM^4^ |
| 1 | RSOLAG1IB_10475 | MoCDIP4 | 3216 | 4E-32 | Effector (plant avirulence determinant) | 4,9 | | 88,8 |
| 2 | RSOLAG1IB_10266 | pnl1 | 3226 | 1E-72 | pectin lyase | 4,7 | | 16,8 |
| 3 | RSOLAG1IB_1987 | PELA | 179 | 2E-46 | Pectate lyase | 3,2 | | 38,0 |
| 4 | RSOLAG1IB_3777 | MGG_04556 | 881 | 1E-27 | Hypothetical protein | 2,2 | | 108,7 |
| 5 | RSOLAG1IB_2789 | bcpme1 | 1028 | 3E-61 | pectin methylesterase | 2,2 | | 551,2 |
| 6 | RSOLAG1IB_7982 | bcpme1 | 1028 | 7E-53 | pectin methylesterase | 2,1 | | 64,4 |
| 7 | RSOLAG1IB_2788 | bcpme1 | 1028 | 3E-36 | pectin methylesterase | 2,1 | | 36,9 |
| 8 | RSOLAG1IB_2787 | bcpme1 | 1028 | 1E-56 | pectin methylesterase | 2,0 | | 43,5 |
| 9 | RSOLAG1IB_10265 | pnl1 | 3226 | 4E-73 | pectin lyase | 2,0 | | 14,7 |
| 10 | RSOLAG1IB_10292 | tmpL | 2296 | 3E-41 | Intracellular Redox Homeostasis | 1,9 | | 16,2 |
| 11 | RSOLAG1IB_6281 | Srt1 | 2240 | 1E-23 | Sucrose Transporter | 1,8 | | 112,9 |
| 12 | RSOLAG1IB_8826 | Srt1 | 2240 | 3E-48 | Sucrose Transporter | 1,8 | | 47,9 |
| 13 | RSOLAG1IB_5157 | GAS1 | 256 | 2E-27 | Appressorial penetration | 1,8 | | 630,9 |
| 14 | RSOLAG1IB_8394 | Srt1 | 2240 | 2E-21 | Sucrose Transporter | 1,7 | | 26,8 |
| 15 | RSOLAG1IB_4264 | BUF1 | 2022 | 1E-22 | No description | 1,7 | | 224,1 |
| 16 | RSOLAG1IB_9933 | BcBOT1 | 438 | 2E-21 | Cytochrome P450 monooxygenase | 1,6 | | 98,2 |
| 17 | RSOLAG1IB_8356 | LYS9 | 2519 | 6E-76 | Saccharopine dehydrogenase | 1,6 | | 21,9 |
| 18 | RSOLAG1IB_4365 | Mohik8 | 3162 | 9E-77 | Histidine Kinase | 1,5 | | 5,6 |
| 19 | RSOLAG1IB_275 | GAT1 | 2600 | 4E-83 | Candidate Effector genes | 1,5 | | 19,2 |
| 20 | RSOLAG1IB_10258 | pnl1 | 3226 | 8E-69 | pectin lyase | 1,0 | | 34,0 |
| ^1^Gene name, PHI-base identifier and e-values as obtained from a protein BLAST against the PHI-base database. | | | | | | |  |  |
| ^2^Putative functions based on BLAST2GO protein BLAST. | | | | | | |  |  |
| ^3^Values calculated with the DESeq R package as it is implemented in the ReadXplorer software. | | | | | | |  |  |
| ^4^Average RPKM during the experiment calculated over all interaction zones. | | | | | | |  |  |

| **Table I. PHI-base analysis of DEGs upregulated in zone 3 *vs*. 2** | | | | | | |  |  |
| --- | --- | --- | --- | --- | --- | --- | --- | --- |
|  | Genome | Gene name^1^ | PHI^1^ | e-value^1^ | Function description^2^ | Log_2_ | | Average |
|  | Locus |  |  |  |  | Fold change^3^ | | RPKM^4^ |
| 1 | RSOLAG1IB_8355 | PELA | 179 | 9E-52 | Pectate lyase | 3,2 | | 32,2 |
| 2 | RSOLAG1IB_6694 | PELA | 179 | 1E-50 | Pectate lyase | 3,1 | | 22,8 |
| 3 | RSOLAG1IB_11299 | Erl1 | 2147 | 4E-39 | GTPase | 3,1 | | 1,0 |
| 4 | RSOLAG1IB_6135 | Tup1 | 2020 | 1E-27 | Transcription repression | 3,0 | | 0,1 |
| 5 | RSOLAG1IB_6692 | PELA | 179 | 4E-53 | Pectate lyase | 2,9 | | 5,2 |
| 6 | RSOLAG1IB_9345 | MGG_05905 | 2107 | 8E-43 | Phospholipase C | 2,8 | | 33,6 |
| 7 | RSOLAG1IB_5930 | CgOPT1 | 2976 | 3E-109 | Oligopeptide transporters of the OPT family | 2,8 | | 173,5 |
| 8 | RSOLAG1IB_6145 | Tup1 | 2020 | 1E-23 | Transcription repression | 2,3 | | 1,2 |
| 9 | RSOLAG1IB_7513 | MGG_04556 | 881 | 2E-35 | Hypothetical protein | 2,3 | | 2,0 |
| 10 | RSOLAG1IB_7296 | bcpg1 | 1027 | 2E-58 | Endopolygalacturonase | 2,3 | | 15,8 |
| 11 | RSOLAG1IB_6061 | Srt1 | 2240 | 6E-22 | Sucrose Transporter | 2,2 | | 76,5 |
| 12 | RSOLAG1IB_10696 | FET3-1 | 2920 | 3E-70 | Functional ferroxidase | 2,2 | | 6,1 |
| 13 | RSOLAG1IB_5700 | PELB | 222 | 6E-67 | Pectate lyase | 2,1 | | 161,3 |
| 14 | RSOLAG1IB_6993 | CTB6 | 1047 | 7E-22 | oxidoreductase | 2,1 | | 150,8 |
| 15 | RSOLAG1IB_6795 | CHIP6 | 243 | 2E-135 | Sterol glycosyl transferase | 2,1 | | 3,7 |
| 16 | RSOLAG1IB_12659 | Tup1 | 2020 | 1E-35 | Transcription repression | 2,1 | | 2,9 |
| 17 | RSOLAG1IB_8400 | bcpg1 | 1027 | 3E-55 | Endopolygalacturonase | 2,0 | | 8,3 |
| 18 | RSOLAG1IB_5336 | MGG_04100 | 2161 | 7E-23 | Cytokinesis | 1,7 | | 1,8 |
| 19 | RSOLAG1IB_7338 | tmpL | 2296 | 1E-50 | Intracellular Redox Homeostasis | 1,6 | | 0,8 |
| 20 | RSOLAG1IB_4183 | pnl1 | 3226 | 4E-83 | pectin lyase | 1,6 | | 0,8 |
| ^1^Gene name, PHI-base identifier and e-values as obtained from a protein BLAST against the PHI-base database. | | | | | | |  |  |
| ^2^Putative functions based on BLAST2GO protein BLAST. | | | | | | |  |  |
| ^3^Values calculated with the DESeq R package as it is implemented in the ReadXplorer software. | | | | | | |  |  |
| ^4^Average RPKM during the experiment calculated over all interaction zones. | | | | | | |  |  |

# Supplementary Chapter 2

# Results and discussion of Carbohydrate-Active enZYmes

Wibberg et al. previously discussed the Carbohydrate active enzymes (CAZy) repertoire as deduced from the *R. solani* AG1-IB 7/3/14 transcriptome [15]. In phytopathogenic organisms, CAZy enzymes are the key players for the degradation of structural compounds like cellulose, pectin, pectate and lignin [16]. Here we present the analysis of CAZy enzyme expression at the transcriptional level during interspecific interaction with *L. sativa*. Overall, about one third of the CAZy motifs found within the genome are differentially transcribed during our experiment. This could indicate that only a specific subset of CAZy enzymes is involved in interspecific interactions in general or more specifically in the interspecific interaction with *L. sativa* cv. Tizian. Please note that although only one third is listed as differentially expressed genes (DEGs), this does not indicate that the other CAZy motif containing genes found within the genome are not expressed at all. Comparing the different zones we observed the highest variety of DEGs containing CAZy motifs within zone 2. Interestingly, within zone 3, based on read counts per kilo base per million reads (RPKM), the largest relative percentage of the total mRNA pool is dedicated to CAZy, the calculation was. This implies that despite the reduced diversity of differentially expressed CAZy enzymes in comparison to zone 2, CAZy enzymes still occupy a major role regarding importance within interaction zone 3. The lowest variety and abundance of CAZy motif containing transcripts is found within zone 1. Compared to the *R. solani* AG1-IB 7/3/14 genomic potential, the classes of GlycosylTransferases (GT) and Carbohydrate-Binding Modules (CBM) are remarkably unrepresented within the DEGs found between the different zones. Different host plants provide the pathogen with substrates of variable carbohydrate compositions, requiring a variety of binding and transferase motifs. Therefore these two CAZy classes probably include members that are differentially regulated in specific pathosystems, thereby regulating host selectivity.

Several CAZy families are represented by more than 10 differentially expressed members within this experiment, these are the families of: Auxiliary Activities AA3 with 15 members, AA9 with 18 members, Carbohydrate-Binding Module CBM1 with 14 members, Glycoside Hydrolases GH28 with 11 members, GH43 with 10 members, Carbohydrate Esterases CE10 with 11 members and Polysaccharide Lyases PL1 with 11 members. Members of these families are involved in binding of cellulose and the degradation of a variety of carbohydrate compounds including: cellobiose, cellulose, pectin, pectate, arabinan and xylose substrates.

A few of the CAZy motif containing genes stand out in particular for being both, highly differentially expressed as well as for featuring a high overall expression level. Most notably are some members of the group of CBM13 family, previously discussed in the Ricin-like lectin chapter. Transcript RSOLAG1IB_10881 encoding the CBM13 motif approximately accounted for 3% of the mRNA pool, based on calculation of RPKM (Figure S3). Another remarkable transcript is RSOLAG1IB_2430 containing a AA6 motif putatively annotated as a WrbA type NAD(P)H quinone oxidoreductase [17] that exponentially increased in its abundance between zone 1 and 3 and has a probable function in phenolic compound degradation.

The transcript of RSOLAG1IB_10475 contains the CAZy AA9 motif with supposed activity in lignocellulose hydrolysis, formerly known as the Glycoside hydrolase 61 motif (GH61). The corresponding gene is overexpressed within zone 1 compared to zone 2, although not significantly and its expression is reduced even further towards zone 3 with a significant 33-fold downregulation. In the fungal plant pathogenic *Colletotrichum* species, this enzyme is coupled to the lifestyle transition from biotrophic to necrotrophic [18]. For *R. solani* AG1-IB a true biotrophic stage has not yet been observed and therefore it makes sense to measure early expression of AA9 during the infection process. In total, 18 genes with AA9 motifs were found to be differentially expressed between the different zones.

Finally the group of differentially expressed O-linked N-acetylglucosamine (OGT) genes that we discussed previously (chapter; Differentially expressed genes between zone 2 and zone 3) have not detected by the CAZy analysis.

Fig B-E provide an overview of the differentially expressed transcripts containing CAZy motifs that were found within this experiment.


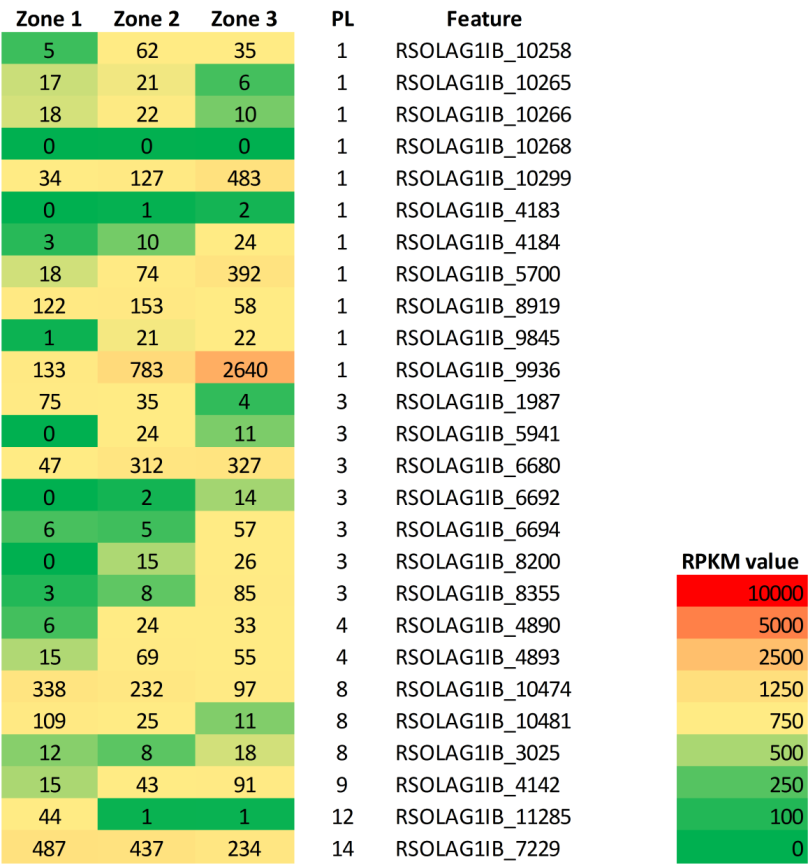


**Figure S1:** Heat map of putative CAZy Polysaccharide-Lyases family transcripts (PL) based on the average RPKM values per interaction zone. Only transcripts with at least one significant upregulation within the experiment as determined by DESeq with a minimum absolute log2 fold change of 1 and a maximum p-adjusted value of 0.05


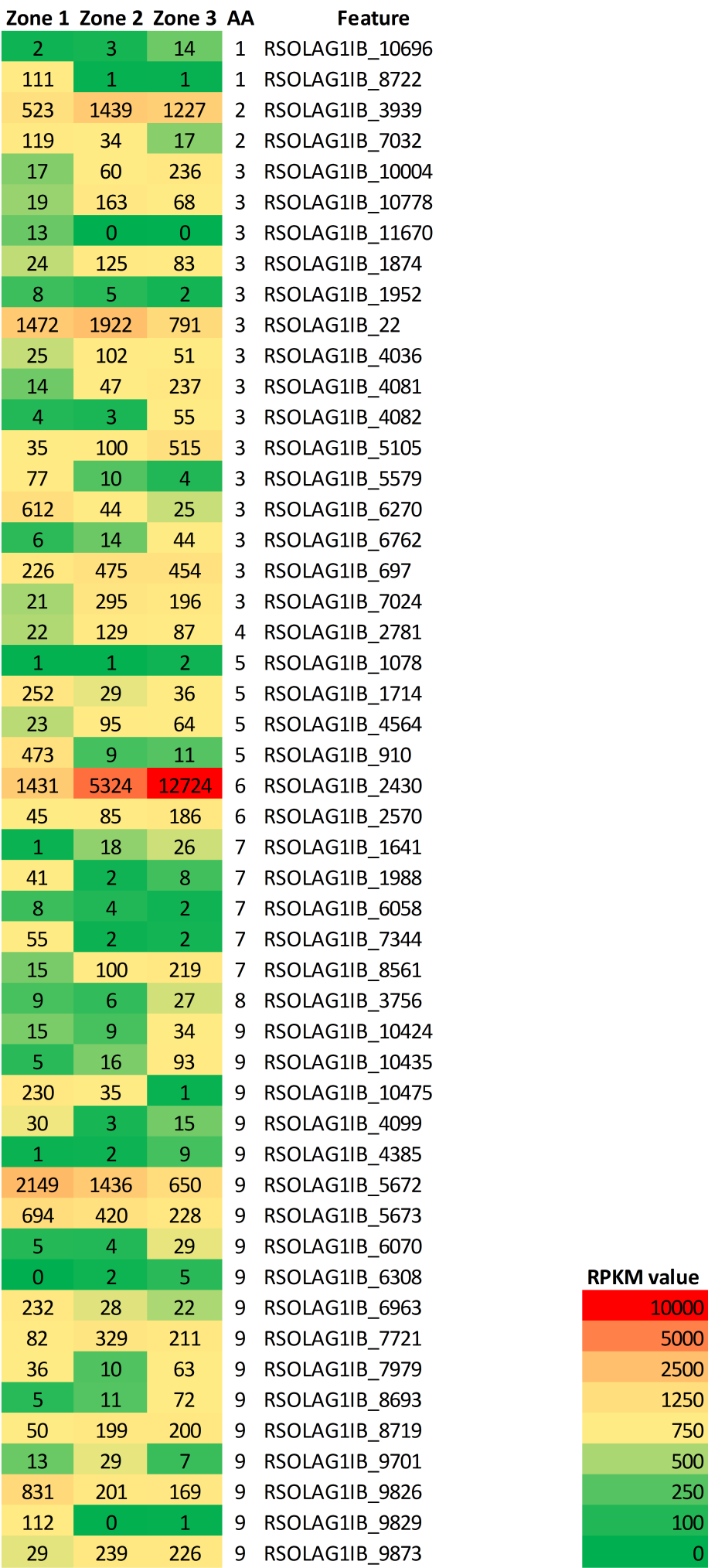


**Fig B.** Heat map of putative CAZy Auxiliary Activities family transcripts (AA) based on the average RPKM values per interaction zone. Only transcripts with at least one significant upregulation within the experiment as determined by DESeq with a minimum absolute log2 fold change of 1 and a maximum p-adjusted value of 0.05


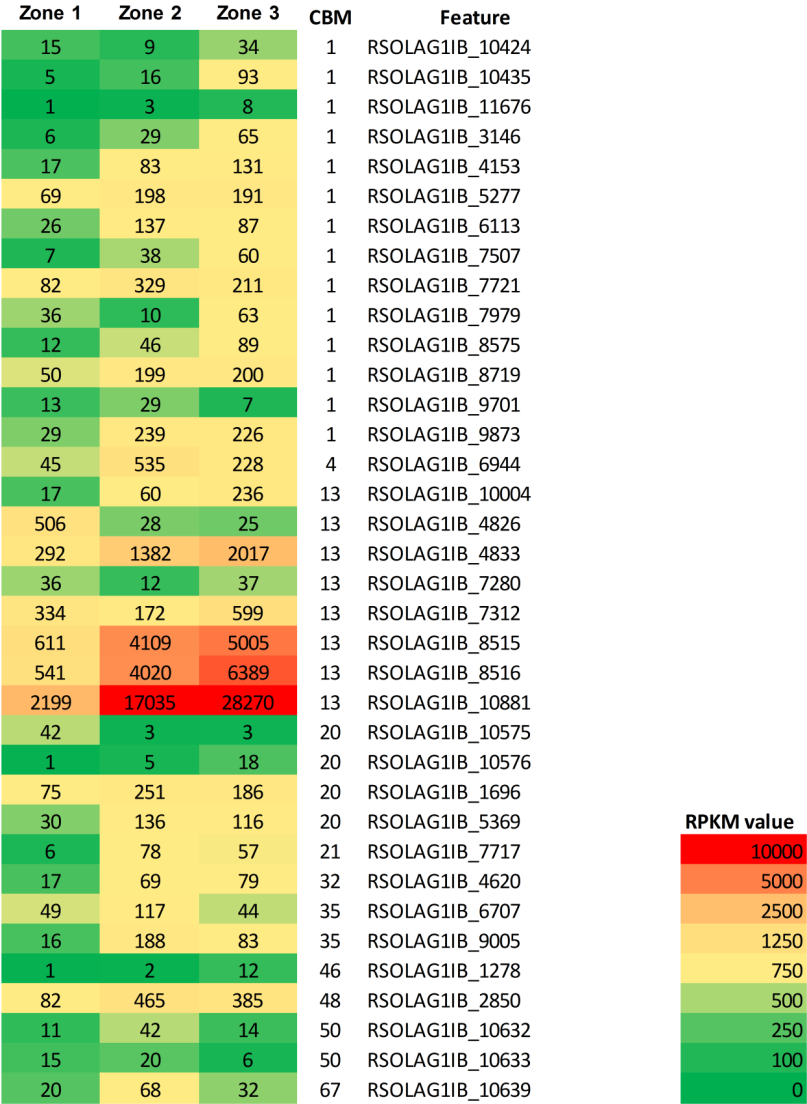


**Fig C.** Heat map of putative CAZy Carbohydrate-Binding Module family transcripts (CBM) based on the average RPKM values per interaction zone. Only transcripts with at least one significant upregulation within the experiment as determined by DESeq with a minimum absolute log2 fold change of 1 and a maximum p-adjusted value of 0.05


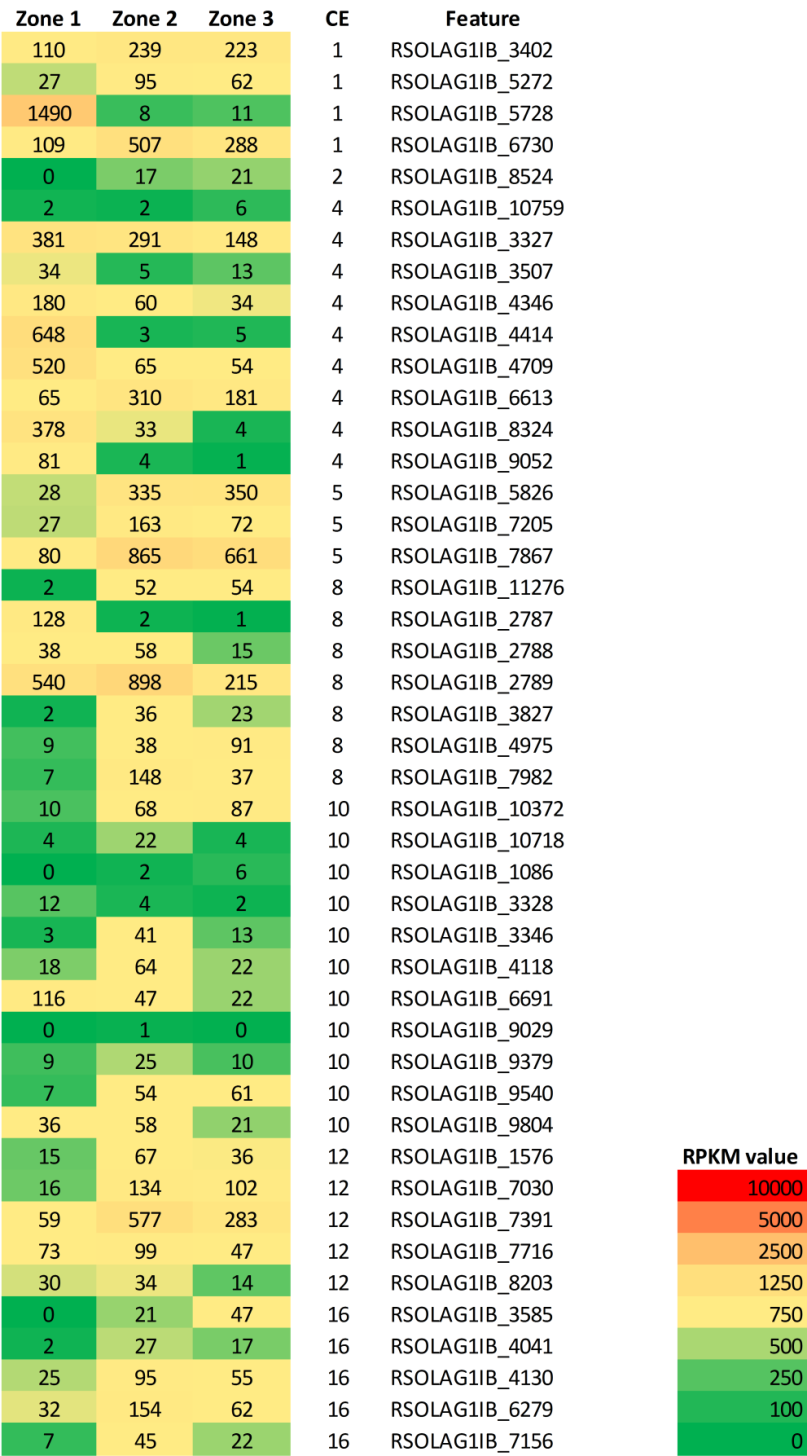


**Fig D.** Heat map of putative CAZy Carbohydrate Esterase family transcripts (CE) based on the average RPKM values per interaction zone. Only transcripts with at least one significant upregulation within the experiment as determined by DESeq with a minimum absolute log2 fold change of 1 and a maximum p-adjusted value of 0.05


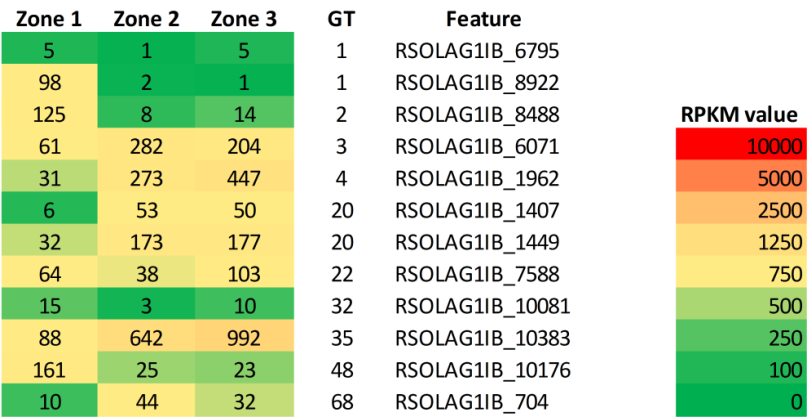


**Fig D.** Heat map of putative CAZy GlycosylTransferase family transcripts (GT) based on the average RPKM values per interaction zone. Only transcripts with at least one significant upregulation within the experiment as determined by DESeq with a minimum absolute log2 fold change of 1 and a maximum p-adjusted value of 0.05


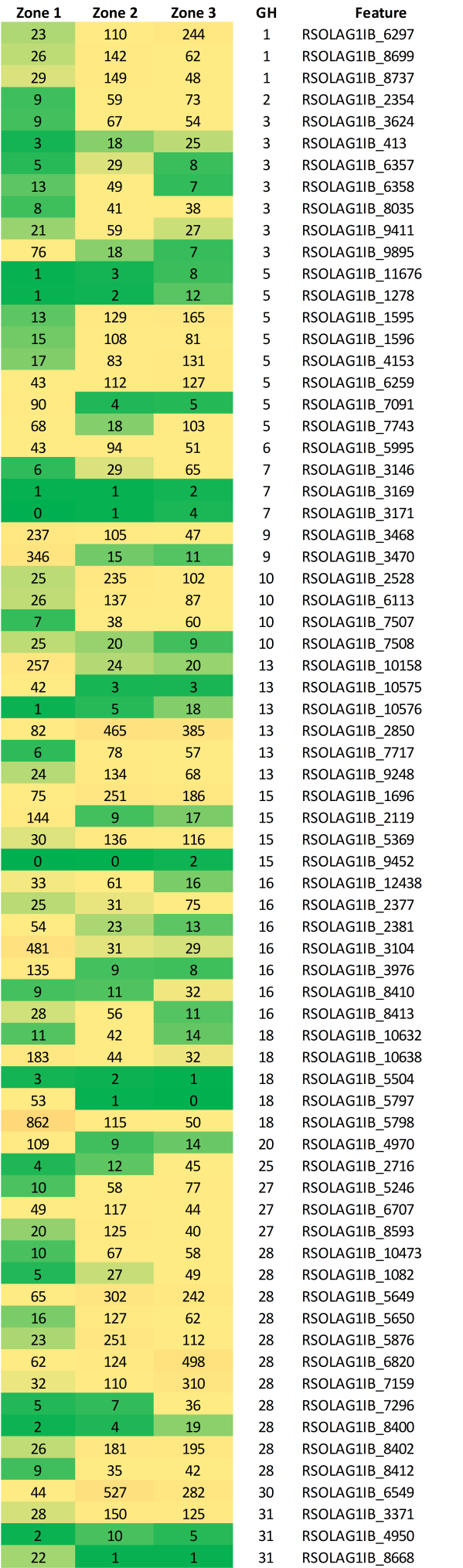

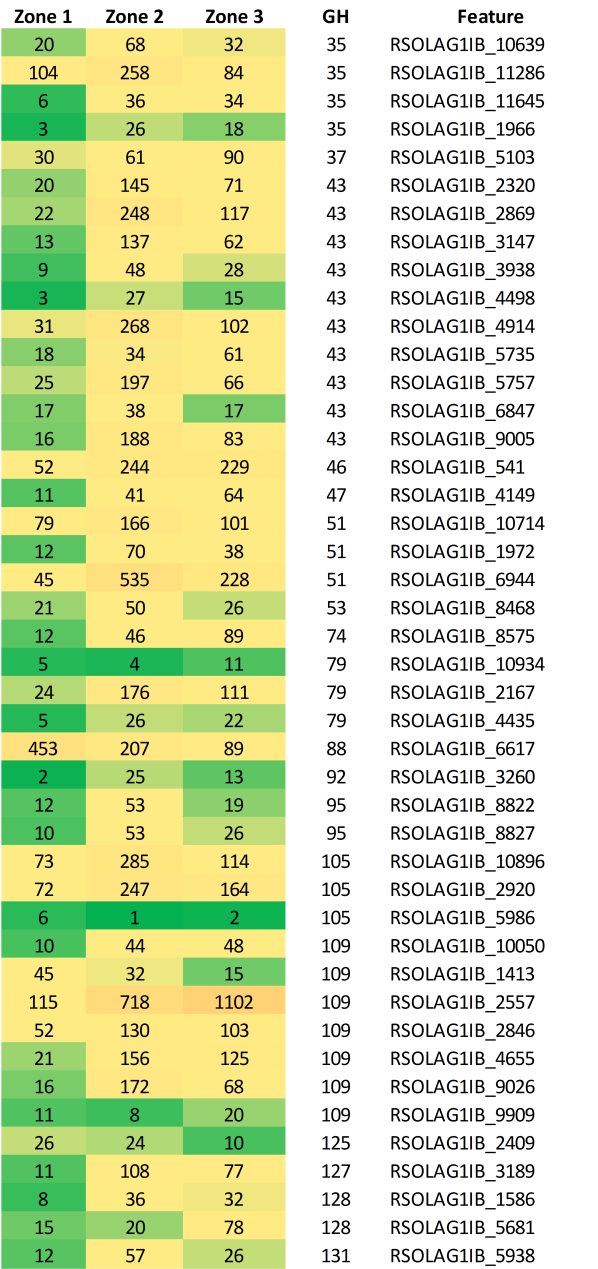

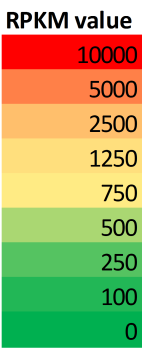


**Fig E.** Heat map of putative CAZy Glycoside Hydrolase family transcripts (GH) based on the average RPKM values per interaction zone. Only transcripts with at least one significant upregulation within the experiment as determined by DESeq with a minimum absolute log2 fold change of 1 and a maximum p-adjusted value of 0.05

# References:

1. Sirim D, Wagner F, Wang L, Schmid RD, Pleiss J. The Laccase Engineering Database: A classification and analysis system for laccases and related multicopper oxidases. Database. 2011;2011: 1–7. doi:10.1093/database/bar006

2. Leuthner B, Aichinger C, Oehmen E, Koopmann E, Müller O, Müller P, et al. A H2O2-producing glyoxal oxidase is required for filamentous growth and pathogenicity in Ustilago maydis. Mol Genet Genomics. 2005;272: 639–650. doi:10.1007/s00438-004-1085-6

3. Patkar RN, Suresh A, Naqvi NI. MoTea4-mediated polarized growth is essential for proper asexual development and pathogenesis in Magnaporthe oryzae. Eukaryot Cell. 2010;9: 1029–38. doi:10.1128/EC.00292-09

4. Berndt P, Lanver D, Kahmann R. The AGC Ser/Thr kinase Aga1 is essential for appressorium formation and maintenance of the actin cytoskeleton in the smut fungus Ustilago maydis. Mol Microbiol. 2010;78: 1484–1499. doi:10.1111/j.1365-2958.2010.07422.x

5. Valdés-Santiago L, Cervantes-Chávez JA, Ruiz-Herrera J. Ustilago maydis spermidine synthase is encoded by a chimeric gene, required for morphogenesis, and indispensable for survival in the host. FEMS Yeast Res. 2009;9: 923–935. doi:10.1111/j.1567-1364.2009.00539.x

6. Yi M, Chi M-H, Khang CH, Park S-Y, Kang S, Valent B, et al. The ER chaperone LHS1 is involved in asexual development and rice infection by the blast fungus Magnaporthe oryzae. Plant Cell. 2009;21: 681–695. doi:10.1105/tpc.107.055988

7. Wilson R a, Gibson RP, Quispe CF, Littlechild J a, Talbot NJ. An NADPH-dependent genetic switch regulates plant infection by the rice blast fungus. Proc Natl Acad Sci U S A. 2010;107: 21902–21907. doi:10.1073/pnas.1006839107

8. Foster AJ, Jenkinson JM, Talbot NJ. Trehalose synthesis and metabolism are required at different stages of plant infection by Magnaporthe grisea. EMBO J. 2003;22: 225–235. doi:10.1093/emboj/cdg018

9. Aliferis K a, Jabaji S. FT-ICR/MS and GC-EI/MS metabolomics networking unravels global potato sprout’s responses to Rhizoctonia solani infection. PLoS One. 2012;7: e42576. doi:10.1371/journal.pone.0042576

10. Elías-Villalobos A, Fernández-Álvarez A, Ibeas JI. The general transcriptional repressor Tup1 is required for dimorphism and virulence in a fungal plant pathogen. PLoS Pathog. 2011;7: e1002235. doi:10.1371/journal.ppat.1002235

11. Espagne E, Balhade P, Penin M, Barreau C. HET-E and HET-D Belong to a New Subfamily of WD40 Proteins Involved in Vegetative Incompatibility Specificity in the Fungus Podospora anserina. 2002;

12. Furukawa T, Syono K. Increased Production of IAA by Rhizoctonia solani is Induced by Culture Filtrate from Rice Suspension Cultures. Plant Cell Physiol. 1998;39: 43–48. Available: http://pcp.oxfordjournals.org/cgi/content/abstract/39/1/43

13. Chagué V, Maor R, Sharon A. CgOpt1, a putative oligopeptide transporter from Colletotrichum gloeosporioides that is involved in responses to auxin and pathogenicity. BMC Microbiol. 2009;9: 173. doi:10.1186/1471-2180-9-173

14. Kim K-H, Willger SD, Park S-W, Puttikamonkul S, Grahl N, Cho Y, et al. TmpL, a transmembrane protein required for intracellular redox homeostasis and virulence in a plant and an animal fungal pathogen. PLoS Pathog. 2009;5: e1000653. doi:10.1371/journal.ppat.1000653

15. Wibberg D, Jelonek L, Rupp O, Kröber M, Goesmann A, Grosch R, et al. Transcriptome analysis of the phytopathogenic fungus Rhizoctonia solani AG1-IB 7/3/14 applying high-throughput sequencing of expressed sequence tags (ESTs). Fungal Biol. 2014;118: 800–813. doi:http://dx.doi.org/10.1016/j.funbio.2014.06.007

16. Lombard V, Golaconda Ramulu H, Drula E, Coutinho PM, Henrissat B. The carbohydrate-active enzymes database (CAZy) in 2013. Nucleic Acids Res. 2014;42: D490–D495. doi:10.1093/nar/gkt1178

17. Patridge E V, Ferry JG. WrbA from Escherichia coli and Archaeoglobus fulgidus Is an NAD ( P ) H : Quinone Oxidoreductase. 2006;188: 3498–3506. doi:10.1128/JB.188.10.3498

18. O’Connell RJ, Thon MR, Hacquard S, Amyotte SG, Kleemann J, Torres MF, et al. Lifestyle transitions in plant pathogenic Colletotrichum fungi deciphered by genome and transcriptome analyses. Nat Genet. Nature Publishing Group; 2012;44: 1060–5. doi:10.1038/ng.2372
